# The golden Syrian hamster (*Mesocricetus auratus*) as a model to decipher relevant pathogenic aspects of sheep-associated malignant catarrhal fever

**DOI:** 10.1177/03009858251315115

**Published:** 2025-02-11

**Authors:** Rosalie Fabian, Eleanor G. Bentley, Adam Kirby, Parul Sharma, James P. Stewart, Anja Kipar

**Affiliations:** 1 University of Zurich, Zurich, Switzerland; 2 University of Liverpool, Liverpool, UK

**Keywords:** animal model, experimental infection, malignant catarrhal fever, ovine gammaherpesvirus-2, pathogenesis, Syrian golden hamster

## Abstract

Malignant catarrhal fever (MCF) is an often fatal, sporadic gammaherpesvirus-induced disease of ruminants with global relevance. Ovine gammaherpesvirus-2 (OvHV-2), with sheep as its reservoir host, is a major cause of MCF in susceptible species. Despite extensive research on the molecular aspects of the disease, its pathogenesis is not yet fully understood. The present study re-established the Syrian golden hamster (*Mesocricetus auratus*) as an amenable animal model of MCF and applied complementary in situ approaches to confirm recent findings in natural disease that could shed new light on pathogenic aspects of MCF. These showed that systemic OvHV-2 infection is associated with T-cell and macrophage-dominated mononuclear infiltrates and vasculitis in various organs. Both T-cells and monocytes/macrophages harbor the virus, and infected leukocytes are abundant in the infiltrates. The results also indicate that OvHV-2 has a broader target cell spectrum, including vascular endothelial cells and selected squamous epithelia. The former supports the interpretation that the inflammatory processes develop due to circulating, activated, infected T-cells and monocytes that home to tissues and emigrate from vessels prone to leukocyte emigration, possibly with direct interaction between virus-infected leukocytes and endothelial cells. The latter supports the hypothesis of graft versus host disease scenario, without viral cytopathic effect on epithelial cells but infiltration of the mucosa by infected T-cells and macrophages. The disease processes are accompanied by evidence of expansion of the T-cell compartments and the monocyte/macrophage pool in lymphatic tissues and bone marrow.

Malignant catarrhal fever (MCF) is a generally fatal disease caused by a group of ruminant gammaherpesviruses (subfamily Gammaherpesvirinae, genus *Macavirus*).^20^ Sheep-associated MCF (SA-MCF), caused by ovine herpesvirus 2 (OvHV-2; formally *Macavirus ovinegamma2*),^17,20,60,72^ occurs worldwide in a wide range of susceptible hosts, including cattle, bison, deer, and occasionally pigs and goats.^2,11,39,42^ Alongside wildebeest-associated (WA-)MCF, SA-MCF is the most relevant form of the disease, and the clinical signs and pathological changes are similar regardless of the specific MCF virus involved.^61,76^

OvHV-2 is endemic in sheep, which represent the natural reservoir host (so-called “host species”).^36^ Sheep are known as unaffected carriers of OvHV-2 and usually harbor the virus in circulating lymphocytes and/or the epithelium of the respiratory tract.^27,74^ However, they are not entirely resistant to SA-MCF and can develop MCF-like disease, both after natural infections and experimental exposure to very high viral inoculation doses.^18,36,37,55,56,74^

The pathological changes in MCF are well known and similar in both cattle and bison, a species in which MCF has become of high economic relevance.^6,51^ However, while being extensively examined and reported, they are most comprehensively described in a widely read textbook.^21,51,52,66,76^ MCF is characterized by a combination of pathological processes, with lymphoid hyperplasia, disseminated mononuclear vasculitis, and erosive/ulcerative mucosal lesions being the most relevant (Supplemental Table S1).^21,51,52,66,76^

It is widely accepted that T-cells, particularly cytotoxic T-cells, play a major role in SA-MCF and dominate the inflammatory infiltrates observed in affected cattle,^44,61,63^ although some studies have shown an equal contribution of monocytes/macrophages.^49,63^ Similarly, circulating T-cells were found to carry the virus in animals with MCF,^54,68^ but also in (experimentally) infected sheep where no restriction to one T-cell subtype was detected (both CD2+CD4+ and CD2+CD8+), and even some monocytes (CD14+) were found to be infected.^45^ As part of a recent in-depth study on *rete mirabile* vasculitis in bovine MCF, our group confirmed that virus-infected T-cells and monocytes are both participating in the process.^63^

So far, despite extensive research on the molecular aspects of the disease and its variable clinical presentation, the pathogenesis of MCF is not fully understood. Based on recent knowledge, it is hypothesized that the pathological processes in MCF are based on an abnormal cytotoxic T-cell activity initiating a graft versus host-like reaction that attacks epithelia and arteries.^76^ Interestingly, the concept of a graft versus host-like pathogenesis was already established before the causative virus was identified, more than 40 years ago.^38^

In natural MCF virus infections, the upper respiratory tract and/or the tonsils are the most likely portal of entry, and this route of infection has also been used in experimental settings with large animals and rabbits. The most common practice is to harvest virus from infected animals for subsequent experimental infection. Animals can become infected with OvHV-2 when nebulized with pooled infected sheep nasal secretion containing defined high DNA copy numbers (10^7^ DNA copies).^16,34^ Inoculation with infected lymphoid cells is an alternative option and can use intravenous or intraperitoneal routes.^11,16,33,34,58,65^ In vitro propagation of OvHV-2 is only possible in the established lymphocyte cell line BJ1035, an immortalized anergic T-cell line originating from a clinical cattle MCF case.^4,7,13,20,46,57,64,65,71^ Such studies provided much of what is currently known about OvHV-2 infection and disease development in deer, cattle, and bison.^13^

Due to practicality and affordability, small animal models allow for more in-depth research into the pathogenesis of the disease and modulation and monitoring of host immune responses, which likely play a relevant role in the susceptibility and development of MCF.^8,35^ Since the first reports in the late 1980s,^9,10^ the rabbit has been established and progressively promoted as a model for SA- and WA-MCF and has also been used in recent years for vaccine development.^12–14,65^

Previously reported findings in the rabbit are summarized in Supplemental Table S1. Pathological descriptions of the disease in rabbits vary in detail but consistent changes include lymphocyte/lymphoid cell/ T-cell infiltrations in liver (portal areas), lungs (perivascular, peribronchial, peribronchiolar, and interstitial), and kidneys (perivascular and interstitial), and arteritis-phlebitis in liver and lungs. Similar infiltrates have been occasionally reported in other organs/tissues,^4,13,16,34,65^ and necrotic or granulomatous hepatitis and lymphadenitis have also been observed.^13,51^ Lymphatic tissues (lymph nodes, spleen, and appendix) have been described as hyperplastic, affecting the T-cell zones.^4,10,16,34,65^ Ulcerative lesions were reported in the tongue and cecum,^13^ and subepithelial infiltrates were reported in the esophagus.^65^ The brains and eyes have either not been examined histologically or were found to be unaffected.^13^ While the rabbit model reflects natural MCF in many aspects, it is relatively resistant to OvHV-2 infection and requires a lethal dose 2-fold higher than, for example, a bison.^15,67^

The hamster has been used as an animal model to study human diseases for over 60 years.^47^ It has served as a model species for experimental infections with more than 70 different viruses, including paramyxoviridae, filoviridae, arenaviridae, phleboviruses, and flaviviruses, with a rising trend in their use.^47^ In particular, during the last 4 years, hundreds of studies used the hamster as model for COVID-19.^26^ The hamster has also been proposed as suitable to model MCF.^8,30,58^ An initial experiment published in 1981 reported inoculation of newborn hamsters with 5x10^3^ TCID_50_ (50% tissue culture infectious dose) “malignant catarrhal disease virus” isolated from African wildebeest calves with MCF.^30^ This was followed a few years later by a more thorough examination where, after intraperitoneal inoculation with lymph node cells isolated from a red deer and a cow with MCF that were passaged several times in hamsters,^8,28^ animals became clinically ill and developed histological changes that appeared similar to those described in cattle with MCF.^8^ Since then, this animal model has not been pursued further.

The present study aimed to re-establish the hamster model of MCF and to extend the characterization of the model by using modern molecular techniques. To do this, Syrian golden hamsters (*Mesocricetus auratus*) were intraperitoneally inoculated with OvHV-2 carrying lymphoid cells from a hamster, clinically monitored, and euthanized at clinical endpoints. After confirmation of systemic infection by PCR, histological and immunohistochemical examinations and RNA in situ hybridization served to characterize the pathological processes and identify viral target cells.

## Material and Methods

### Cell Culture and Virus

In an initial experiment, OvHV-2 infected hamster lymphoid cells were kindly provided by Dr George Russell (Moredun Institute, Edinburgh, UK). The cells were from a previous experiment undertaken in 2005 where hamsters were experimentally infected with sheep-associated OvHV-2 and euthanized when they presented with either loss of appetite, depression, diarrhea, and/or ocular/nasal discharge. From the hamsters of this pilot experiment, spleen and lymph nodes were collected and passed through a 70 µm cell strainer (Corning, Corning, USA), and the cells collected were stored in liquid nitrogen until their use in the following larger experiment.

Prior to infection, cells were retrieved from liquid nitrogen then live dead sorted using the EasySep Dead Cell Removal (Annexin V) Kit (Stemcell Technologies, Vancouver, Canada) according to the manufacturer’s instruction. Live cells were adjusted to 1x10^6^ cells/ml and stored on ice.

### Hamsters

Animal work was approved by the local University of Liverpool Animal Welfare and Ethical Review Body and performed under UK Home Office Project License PP4715265. Male 8 to10-week-old golden Syrian hamsters were purchased from Janvier Labs (France). Hamsters were maintained under specific pathogen-free barrier conditions in individually ventilated cages and fed pelleted food ad libitum with free access to water. Prior to experimentation hamsters were acclimatized to their cage for 1 week.

### Experimental Infections

In both the initial study (n = 4) and the subsequent main study (n = 6), animals were inoculated intraperitoneally with pooled spleen and lymph node cells from an OvHV-2 infected hamster. In the first experiment, each animal received 2.2 × 10^5^ cells, which was estimated to be 1.5 × 10^8^ copies/dose. In the second experiment, the animals were inoculated with 1.3 × 10^5^ cells per hamster, which was estimated to be 1.2 × 10^6^ copies/dose. All animals were closely monitored after inoculation for adverse effects including weight loss, hunching, isolation, and ruffled fur. Body temperatures were monitored daily with a subcutaneous temperature transponder (Plexx, Netherlands), and health conditions were monitored daily.

Infection was defined as established when an animal sustained an increase of 1 °C in its average body temperature (defined as the average of the first 5 days recorded) for over 48 h.^46^ Other clinical signs included but were not limited to facial grimace (orbital tightening, bulging of nose or cheek, and dropped ears and/or whiskers), scruffy coat, diarrhea, and swollen feet. Once the infection was clinically confirmed, animals were euthanized by a Schedule 1 method and immediately dissected after death.

For the animals in the initial study, blood samples were collected under terminal anesthesia into EDTA-containing vacutainers (Becton Dickson, Wokingham, UK) and centrifuged at 800× *g* for 10 min. The buffy coat was collected for DNA extraction and quantitative PCR. A selection of organs suspected to exhibit pathological changes based on the clinical signs was collected and fixed in 10% buffered formalin.

From the animals in the main experiment, all major organs as well as the entire body were fixed in 10% buffered formalin for histological examination; after 48 h fixation, the tissues were transferred to 70% ethanol until processing.

Tissues from uninfected animals from another unrelated study served as controls, in particular for the assessment of the hemolymphatic tissues.

### DNA Extraction and Quantification

Buffy coat cells were homogenized and DNA was extracted using the DNeasy Blood & Tissue Kit (Qiagen, Manchester, UK) according to the manufacturer’s instructions. The extracted DNA was quantified using a Nanodrop one spectrophotometer (Thermo Fisher, Waltham, US).

### Quantitative Polymerase Chain Reaction for Ovine Gammaherpesvirus-2

For quantitative polymerase chain reaction (qPCR), TaqMan Universal PCR Master Mix (Applied Biosystems) was used in 20 µl reaction volumes. Each mix consisted of 10 µl TaqMan Universal PCR Master Mix (2X), 2 µl each of forward and reverse primers (100 nM), 2 µl probe (250 nM) for either OvHV-2 or *18s ribosomal* DNA, and 100 ng template DNA suspended in 4 µl nuclease-free water. OvHV-2 and *18s* primers were as described in previous studies.^59,69^ Detection of the *18s ribosomal* DNA internal genome served to normalize for DNA variability and contaminants to give viral copies/µg. The real-time PCR was run with the following cycling conditions: 50 °C for 2 min, 95 °C for 10 min, then 39 cycles of 95 °C for 15 sec and 60 °C for 1 min. The data were collected during step 4 of the PCR program.

### Histology, Immunohistochemistry, and RNA In Situ Hybridization

From animals in the initial study, a selection of organs (skin, tongue, stomach, small intestine, liver, spleen, lungs, kidneys, and the brain from 3 animals, lung from the fourth) was processed. From all animals in the main experiment, samples from the tongue, esophagus, stomach, small intestine, liver, spleen, lymph nodes (cervical, mediastinal, mesenteric), heart, trachea, lungs, kidneys, brain, lumbar spinal cord (cross-section and longitudinal section of 3-5 mm length), right sciatic nerve, and the *M. biceps femoris* were processed. The tissue samples were trimmed and routinely embedded in paraffin. Consecutive sections (3-4 μm thick) were prepared and routinely stained with hematoxylin and eosin and, in selected specimens, subjected to immunohistochemistry (IHC), and RNA in situ hybridization (RNA-ISH).

Lymphatic tissues (mediastinal lymph nodes and spleen) from age-matched, mock infected control hamsters of an unrelated COVID-19 study served for the comparative assessment with the tissues from the infected hamsters in the present study.

IHC was used to characterize the infiltrating leukocytes, including T-cells (CD3+), B-cells (CD79a+), and macrophages (IBA1+), and highlight proliferating cells (PCNA+) and apoptotic cells (cleaved caspase 3+) using cross-reactive antibodies and previously established protocols.^23,26,43,53,70^ All antibodies and detection methods are listed in Supplemental Table S2. Immunohistochemistry assays were carried out in an autostainer (“Link 48” [Agilent Dako]) using the horseradish peroxidase method. Briefly, sections were deparaffinized through graded alcohol and subjected to antigen retrieval in citrate buffer (pH 6.0) or Tris/EDTA buffer (pH 9) for 20 min at 98 °C. Slides were subsequently incubated with the primary antibodies diluted in antibody diluent (Dako). This was followed by blocking of endogenous peroxidase (peroxidase block, Dako) for 10 min at room temperature and incubation with the appropriate secondary antibodies/detection systems following the manufacturers’ protocols. Sections were washed with phosphate-buffered saline (pH 8) between each incubation step. They were counterstained with hematoxylin for 20 s and mounted. The lymph node from an uninfected control hamster served as a positive control for all markers; sections incubated without the primary antibodies served as negative controls.

RNA-ISH was performed using the RNAscope ISH method (Advanced Cell Diagnostics (ACD), Newark, California), Ov2.5 mRNA (coding for OvHV-2 viral IL-10; Genbank NC_007646.1) oligoprobes, and the automated RNAscope 2.5 Detection Reagent Kit (brown) according to the manufacturer’s protocol, and as previously published, applying slight modifications.^42,63^ Briefly, sections were heated to 60 °C for 1 h, deparaffinized and permeabilized by incubation in pretreatment solution 1 (RNAscope Hydrogen Peroxide) for 10 min at room temperature, followed by boiling in RNAscope 1X Target Retrieval Reagents solution at 100 °C for 25 min and washing in distilled water and ethanol. This was followed by digestion with RNAscope Protease Plus for 30 min, hybridization with the oligoprobes for 2 h, and serial amplification with different amplifying solutions (AMP1, AMP2, AMP3, and AMP4: alternating 15 min and 30 min), all at 40 °C in a humidity control tray for 2 h (HybEZ Oven, ACD). Between each incubation step, slides were washed for 2 × 2 min with washing buffer. They were subsequently incubated with AMP 5 (45 min), AMP 6 (15 min), and diaminobenzidine (10 min) at room temperature in the humidity control tray. Sections were counterstained for 15 s with Gill’s hematoxylin, then dehydrated with graded alcohol and xylene and coverslipped. A formalin-fixed, paraffin-embedded cell pellet of BJ1035 cells (permanently OvHV-2 infected bovine large granular lymphocyte cell line) served as a positive control.^63^ Consecutive sections incubated accordingly but without including the hybridization step and sections from a formalin-fixed, paraffin-embedded liver sample of a hamster not infected with OvHV-2 served as negative controls.

In order to determine the leukocytes that carry the virus, a combined RNA-ISH/IHC protocol was applied to sections of the liver of case 10. As a first step, IHC for CD3 and IBA1 was undertaken after the ISH pretreatment steps (baking, deparaffinizing, incubating in RNAscope Hydrogen Peroxide, cooking in RNAscope 1X Target Retrieval Reagents solution, and incubating in RNAscope Protease Plus) instead of the normal antigen retrieval process for IHC. Since the immunolabeling was maintained after this pretreatment, further sections were subjected to the entire RNA-ISH protocol, without letting the sections dry at any time point during the procedure. Subsequently, the sections were incubated in tap water instead of counterstaining with hematoxylin and dehydration, followed by the respective IHC protocol, with overnight incubation of the antibodies at the dilutions indicated in Supplemental Table S2. The reaction was visualized with the same kit as indicated in Supplemental Table S2 but with AEC single solution (Zytomed Systems GmbH, Berlin, Germany) instead of diaminobenzidine, followed by counterstaining for IHC.

## Results

### Clinical Findings and Viremia

In an initial study, 4 hamsters were inoculated intraperitoneally with spleen and lymph node cells derived from infected hamsters. The first hamster (case 1) developed a sustained elevated temperature and further clinical signs (swollen left hind limb, limping) by 29 days post-inoculation (dpi), the second appeared healthy but was euthanized at 33 dpi due to an elevated body temperature, and the remaining 2 hamsters (cases 3 and 4) were euthanized at 41 dpi due to elevated body temperatures (Supplemental Table S3). One (case 3) had also developed a facial grimace and scruffy coat, and the other (case 4) developed diarrhea. In all 4 animals, the buffy coat was positive for OvHV-2 DNA by qPCR, with 94 to 1.5 × 10^5^ copies/µg, confirming that the animals were viremic at the time of death.

In the second experiment (6 hamsters), the clinical course was more rapid. Animals were euthanized at 15-17 dpi following a rise in body temperature and presentation of general malaise and a combination of other clinical signs including piloerection and facial grimace (Supplemental Table S3). The postmortem examination did not reveal any gross changes in any animal, apart from evidence of mild subcutaneous edema in the hamster with a swollen hindleg (case 1) and the presence of coagulated blood in the urinary bladder of case 6.

### Histologic and Immunohistochemical Characterization and Viral Localization of Lesions

Animals in both experiments exhibited consistent changes in a range of organs, although the changes often varied in their extent. Information on affected organs is provided in Supplemental Table S3. Subsequently, the main findings are reported.

#### Liver (n = 9)

**Figure 1.**
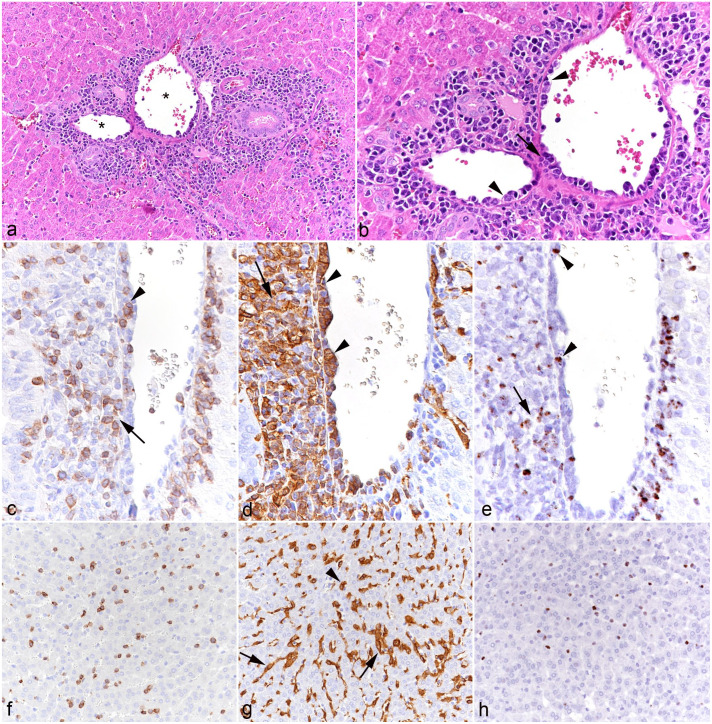
Liver, case 10. (a) Portal area with moderate mononuclear infiltration. Asterisks: portal veins with evidence of vasculitis. Hematoxylin and eosin (HE). (b) Higher magnification of portal veins with subendothelial aggregates of leukocytes (arrowheads) and infiltration of the vessel wall (arrow) consistent with vasculitis. HE. (c-e) Portal vein. (c) Among the leukocytes in the subendothelial aggregates (arrowhead) and surrounding the vein are numerous CD3+ T-cells. CD3 immunohistochemistry (IHC). (d) The subendothelial (arrowheads) and perivascular (arrow) leukocyte aggregates are dominated by IBA1+ monocytes/macrophages. IBA1 IHC. (e) Among the leukocytes in the subendothelial aggregates (arrowhead) and surrounding the vein (arrow) are several positive for viral RNA (Ov2.5). RNA in situ hybridization (ISH). (f-h) Hepatic sinusoids. (f) Sinusoids contain increased numbers of CD3+ T-cells. CD3 IHC. (g) IBA1 immunolabeling highlights increased numbers of monocytes (arrowhead) in addition to Kupffer cells (arrows). IBA1 IHC. (h) Numerous leukocytes in the sinuses are positive for viral RNA (Ov2.5). RNA-ISH.

All animals exhibited portal, predominantly mononuclear, infiltrates, as well as an increase in leukocytes within the sinusoids (Fig. 1). The portal and sinusoidal infiltrates were comprised of T-cells (CD3+) and even more numerous macrophages (IBA1+) of which many were positive for viral RNA (Ov2.5) (Fig. 1c–h). In some animals (cases 7, 8, and 10), the inflammatory infiltrates were associated with subendothelial leukocyte aggregation and focal infiltration of the wall in portal veins (Fig. 1b–e), consistent with vasculitis. One hamster (case 3) exhibited small endothelium-attached leukocyte thrombi in one portal vein.

#### Heart (n = 6)

**Figure 2.**
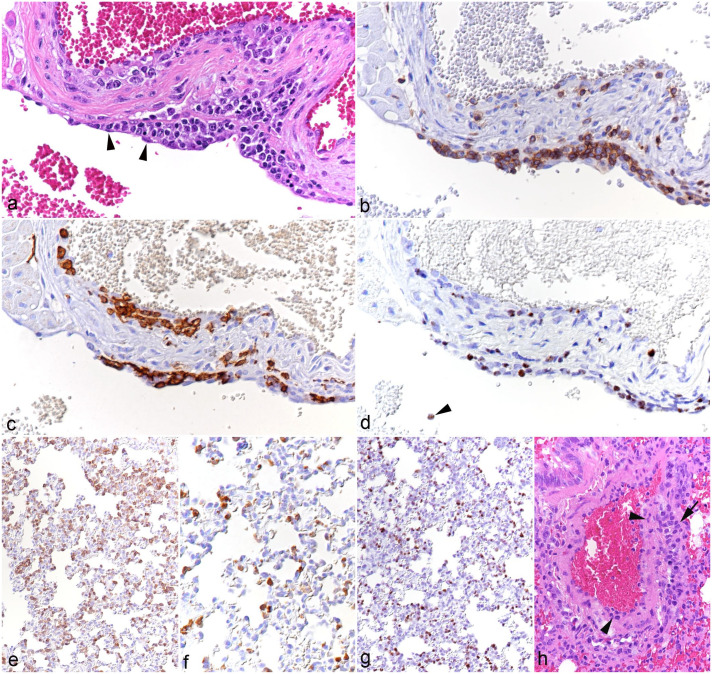
(a-d) Heart, left atrioventricular valve, case 8. (a) Multifocal leukocyte infiltration beneath the endothelium (arrowheads). Hematoxylin and eosin (HE). (b) Among the infiltrating leukocytes are abundant CD3+ T-cells. CD3 immunohistochemistry (IHC). (c) Among the infiltrating leukocytes are abundant IBA1+ monocytes/macrophages. IBA1 IHC. (d) Several infiltrating leukocytes are positive for viral RNA (Ov2.5). Arrowhead: Ov2.5 mRNA positive leukocyte in the blood. RNA in situ hybridization (ISH). (e-h) Lung. (e-g) Case 8. (e) There are abundant CD3+ T-cells within capillaries. CD3 IHC. (f) Capillaries contain increased numbers of IBA1+ monocytes. IBA1 IHC. (g) Abundant leukocytes within capillaries are positive for viral RNA (Ov2.5). RNA-ISH. (h) Case 3. Artery with leukocytes in the wall (arrowheads) and in the surrounding tissue (arrow), consistent with arteritis. HE.

The myocardium and left atrioventricular valves were examined in animals in the main experiment (cases 5-10); these animals all exhibited mild to moderate mononuclear valvular endocarditis, with subendothelial leukocyte aggregates (T-cells and monocytes in comparable proportions), many of which were positive for viral RNA (Fig. 2). In 2 hamsters (cases 7 and 10), the ventricular endocardium contained identical infiltrates. In all animals, the myocardium showed mild multifocal interstitial, partially perivascular T-cell and macrophage infiltrates.

#### Lungs (n = 10)

The most consistent finding in the lungs of all animals was a substantial increase in T-cells and, though less numerous, monocytes within capillaries (Fig. 2e, f); whether a proportion of the cells seen in the alveolar walls were located in the interstitium could not be clearly discerned. Many of the leukocytes were found to harbor viral RNA (Fig. 2g). B-cells (CD79a+) were rare in capillaries. In a few animals, there was evidence of leukocyte rolling and emigration (cases 1, 3, and 4) or mild perivascular leukocyte infiltration (cases 1-5). One hamster (case 3) exhibited mild inflammation of an artery (Fig. 2h).

#### Alimentary tract

**Figure 3.**
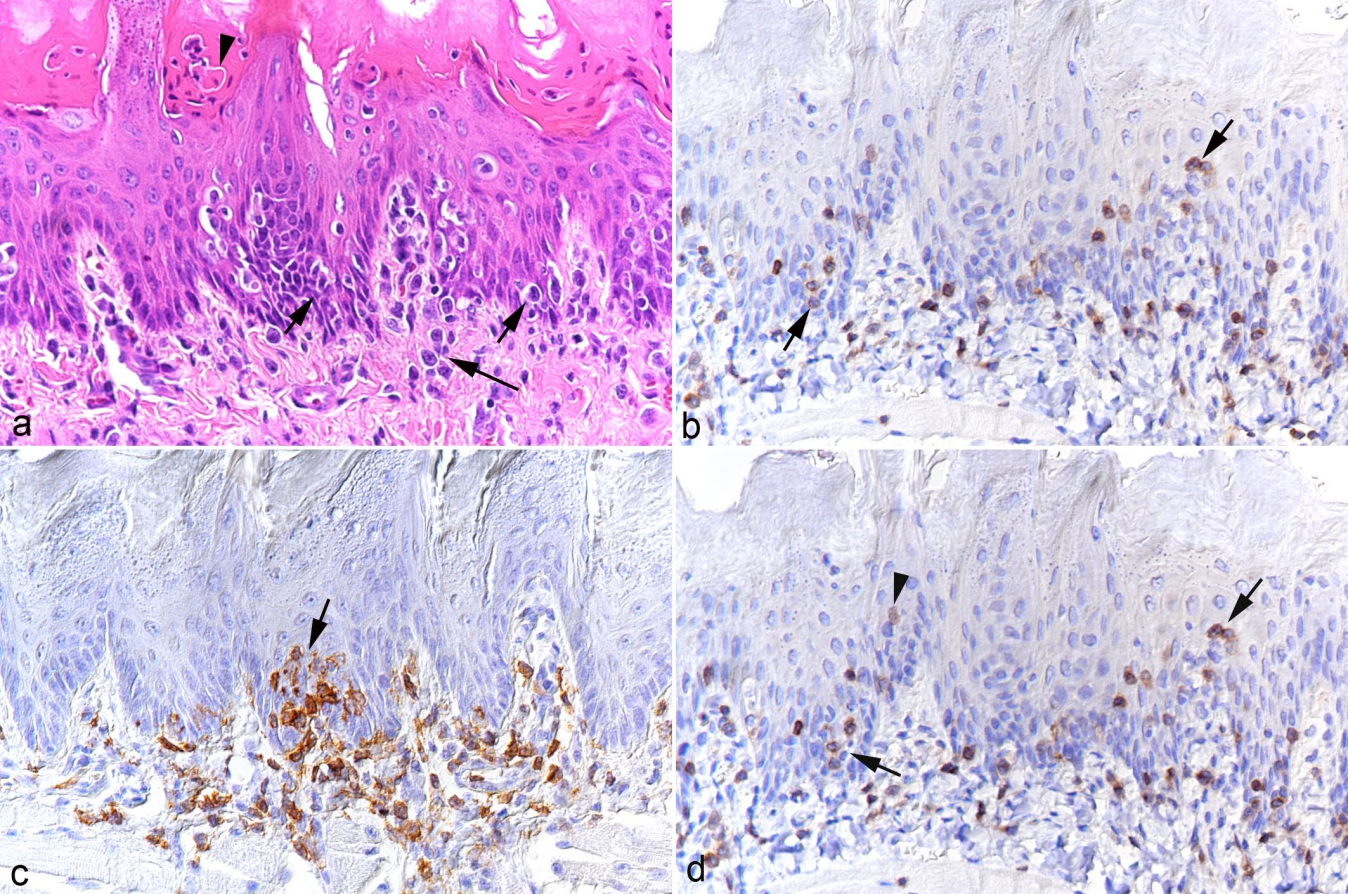
Tongue, case 3. (a) Leukocyte infiltration beneath (long arrow) and within (short arrows) the epithelial layer. There are also individual apoptotic epithelial cells (arrowhead). Hematoxylin and eosin. (b) Infiltrating CD3+ T-cells are beneath the epithelium but are also present between epithelial cells (arrows). CD3 immunohistochemistry (IHC). (c) Focal IBA1+ macrophage-rich subepithelial infiltrate with aggregates of macrophages between epithelial cells (arrow). IBA1 IHC. (d) Abundant infiltrating leukocytes both subepithelial and infiltrating the epithelium (arrows) are positive for viral RNA (Ov2.5). There are also individual cells with nuclear signals that have the morphology of epithelial cells (arrowhead). RNA in situ hybridization.

**Figure 4.**
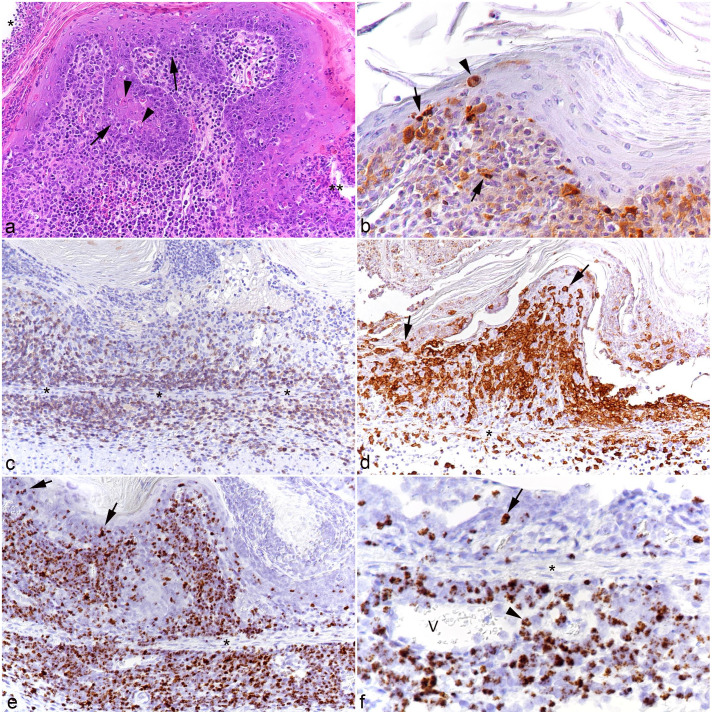
Non-glandular forestomach, case 3. (a) Serocellular crust formation (*) with focal ulceration (**) and marked subepithelial leukocyte infiltration. Leukocytes focally invade the basal epithelial cell layers (arrows). There are individual apoptotic epithelial cells (arrowheads). Hematoxylin and eosin. (b) Cleaved caspase 3 immunolabeling confirms apoptosis of individual epithelial cells (arrowhead) and also shows that some leukocytes both within the epithelium and in the submucosal infiltrate undergo apoptosis (arrows). Cleaved caspase 3 immunohistochemistry (IHC). (c) A large proportion of infiltrating leukocytes in the mucosa and submucosa are CD3+ T-cells. The asterisks highlight the muscularis mucosae. CD3 IHC. (d) Area with extensive serocellular crust formation. There are abundant IBA1+ macrophages in the mucosal infiltrate that also infiltrate the epithelium (arrows). The asterisk highlights the muscularis mucosae. IBA1 IHC. (e, f) RNA in situ hybridization (ISH) for Ov2.5. The asterisks highlight the muscularis mucosae. (e) Abundant leukocytes in the mucosal and submucosal infiltrates and infiltrating the epithelium (arrows) are positive for viral RNA. (f) Higher magnification of a submucosal vein (V) highlights recruitment of viral RNA positive leukocytes (arrowhead). The epithelium contains a cell with a nuclear signal that has the morphology of an epithelial cell (arrow).

The tongue (n = 9) consistently exhibited mononuclear subepithelial infiltrates of varying intensity (Fig. 3). The infiltrates were composed of T-cells and macrophages, which also invaded the epithelium (Fig. 3b, c). In some cases, this was associated with apoptosis of individual epithelial cells (Fig. 4a). With more intense infiltration, inflammatory cells also stretched between the musculature. A proportion of infiltrating leukocytes were found to express viral RNA (Fig. 3d).

In the esophagus (n = 6), similar subepithelial infiltrates were observed. However, these were only focal and mild, with occasional individual leukocytes between basal epithelial cells.

In most animals (cases 2-4 and 7-10), the stomach (n = 9) contained diffuse mild to moderate mononuclear cell infiltrates in the mucosa; in 3 animals (cases 2-4), these extended into the submucosa. Again, the infiltrates were comprised of T-cells and macrophages, with a large proportion of cells positive for viral RNA. In the non-glandular forestomach, infiltrating cells were observed between the epithelial cells in 3 hamsters (cases 5, 6, and 10). Case 3 had severe erosive to ulcerative proventricultis with multifocal serocellular crusts, pustule formation, and abundant apoptotic keratinocytes (Fig. 4a, b; Supplemental Figure S1). There was a marked T-cell and macrophage-dominated submucosal infiltrate that also invaded the basal epithelial layer and harbored abundant virus RNA-positive leukocytes (Fig. 4c-f), with evidence of recruitment of infected leukocytes from the blood (Fig. 4f).

In all animals, small intestinal villi were diffusely expanded due to a marked mononuclear infiltrate dominated by T-cells followed by macrophages, with only rare B-cells and plasma cells (Supplemental Figures S2a-e). Abundant cells in the infiltrate carried viral RNA (Supplemental Figure S2d); the latter was also seen in lymphocytes in the generally prominent Peyer’s patches. The infiltrate was often associated with villus blunting and fusion (Supplemental Figure S2f).

#### Nervous system

**Figure 5.**
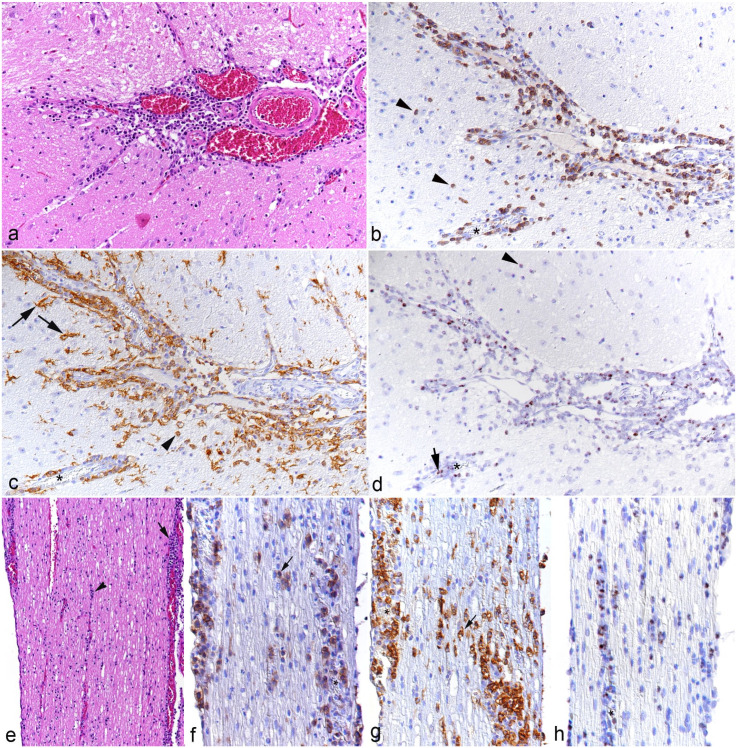
Brainstem and spinal cord, case 10. (a-d) Brainstem. (a) Leptomeninges with moderate perivascular leukocyte infiltration. Hematoxylin and eosin (HE). (b) Numerous leukocytes in the leptomeningeal and parenchymal perivascular infiltrates are CD3+ T-cells. Some of these cells have migrated further in the parenchyma (arrowheads). CD3 immunohistochemistry (IHC). (c) Numerous leukocytes in the leptomeningeal and parenchymal perivascular infiltrates are IBA1+ macrophages. Some of these have migrated further in the parenchyma (arrowhead). The affected areas contain IBA1+ cells with the morphology of activated microglia (arrows). IBA1 IHC. (d) A proportion of the leukocytes in the leptomeningeal and parenchymal perivascular infiltrates are positive for viral RNA (Ov2.5). Some of these have migrated further in the parenchyma (arrowhead). Arrow: Ov2.5 mRNA positive leukocyte in the blood. Asterisk: affected parenchymal vessel. RNA in situ hybridization (ISH). (e-h). Lumbar spinal cord, longitudinal section. (e) Moderate, multifocal leptomeningeal (arrow) and mild, perivascular (arrowhead) leukocyte infiltration. HE. (f, g) Area with intense inflammatory infiltration. (f) CD3+ T-cells are abundant in the perivascular infiltrates, but are also present as individual cells between nerve fibers. CD3 IHC. (g) IBA1+ macrophages are also abundant in the perivascular infiltrates, and there are IBA1+ cells with the morphology of activated microglia (arrows) between nerve fibers. IBA1 IHC. (h) A proportion of the infiltrating leukocytes are positive for viral RNA (*Ov2.5*). RNA-ISH.

In all examined brains (all animals from the main experiment, cases 5-10), there was a mild to moderate, focally extensive to diffuse mononuclear leptomeningitis with distinct perivascular arrangements of the infiltrating cells, which were identified as T-cells and macrophages. With more extensive meningeal infiltrates, involvement of the parenchyma (mononuclear encephalitis) was observed and was represented by perivascular infiltrates of a similar composition (Fig. 5a–c), associated with microglial activation of the surrounding parenchyma, which also contained individual migrating leukocytes. A proportion of the infiltrating leukocytes carried viral RNA (Fig. 5d). Changes consistent with leukocytoclastic vasculitis were seen in all hamsters with encephalitis apart from case 9. Interestingly, the 3 available brains of the animals in the pilot study (cases 2-4) only exhibited scattered T-cells in the leptomeninges; however, some of these cells harbored viral RNA.

In animals from the main experiment (cases 5-10), the lumbar spinal cord, a sciatic nerve, and a biceps femoris muscle were also examined. Mild leukomyelitis and leptomeningitis were consistently observed (Fig. 5e–h). The latter was found to extend to the spinal ganglia (periganglioneuritis) and was comprised of T-cells and macrophages (Fig. 5f, g), with evidence of myelinophages (Fig. 5g), multifocal microgliosis (Supplemental Figure S3), and mild satellitosis in the gray matter. Infiltrating leukocytes often carried viral RNA (Fig. 5h). In 5 of the 6 examined cases, the sciatic nerve exhibited mild focal perineurinal and occasionally perivascular neuronal T-cell and macrophage infiltrates, which contained cells positive for viral RNA (Supplemental Figure S4). The biceps femoris muscle was generally unaltered; however, in 3 animals (cases 5, 7, and 8), it contained mild interstitial, perivascular leukocyte infiltrates.

#### Kidneys (n = 9)

In most animals (cases 2, 3, and 5-10), the kidneys contained focal to multifocal, interstitial, mononuclear infiltrates composed of T-cells and macrophages and/or subepithelial infiltrates in the renal pelvis, consistent with pyelitis. The infiltrates varied in their extent and contained variable proportions of leukocytes positive for viral RNA.

In case 6, the urinary bladder was also examined histologically due to macroscopic changes. Histologically, there was cystitis characterized by mucosal and submucosal heterophilic and lymphohistiocytic infiltrates that extended around the proximal part of the urethra and into the surrounding adipose tissue.

*Skin* (n = 3). Skin from the dorsum was histologically examined in 3 animals from the pilot study (cases 2-4). It did not exhibit any histological changes; hence the skin was not examined in animals in the main experiment.

### Cellular Distribution of Ovine Gammaherpesvirus-2 RNA

**Figure 6.**
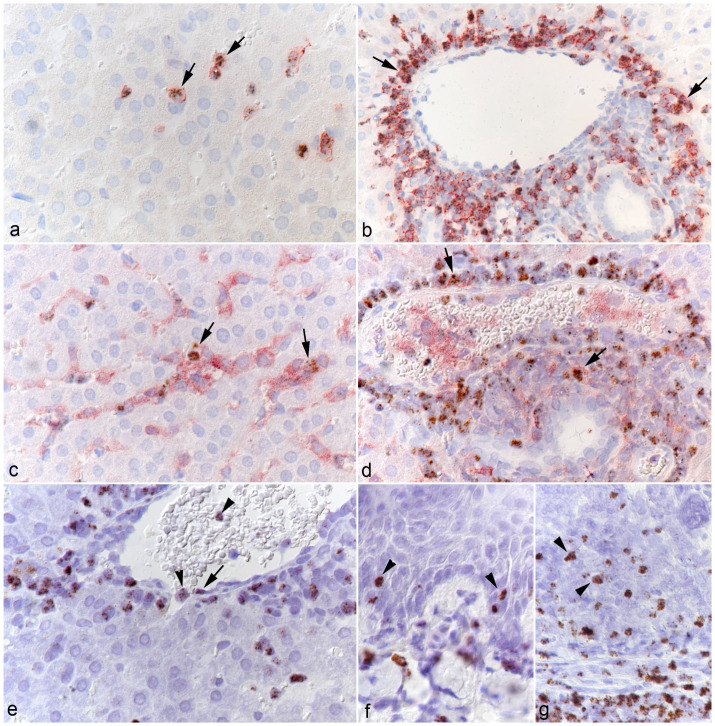
Target cells of OvHV-2 in the hamster. (a-d) Liver, case 10. (a) Several CD3+ T-cells in the sinusoids (arrows) harbor OvHV-2 RNA (*Ov2.5*). RNA in situ hybridization (ISH) and CD3 immunohistochemistry (IHC). (b) Abundant CD3+ T-cells in the (perivenous) portal infiltrates (arrows) harbor OvHV-2 RNA (*Ov2.5*). RNA-ISH and CD3 IHC. (c) Several IBA1+ monocytes in the sinusoids (arrows) harbor OvHV-2 RNA (Ov2.5). RNA-ISH and IBA1 IHC. (d) Few IBA1+ macrophages in the (perivenous) portal infiltrates (arrows) harbor OvHV-2 RNA (Ov2.5). RNA-ISH and IBA1 IHC. (e) Liver, case 6. Central vein with leukocytes in the lumen and the adjacent sinusoid (arrowheads) positive for viral RNA (*Ov2.5*). There is also a cell with the morphology of an endothelial cell that harbors OvHV-2 RNA (arrow). RNA-ISH. (f, g) Case 1. (f) Tongue. A few squamous epithelial cells exhibit a nuclear signal for OvHV-2 RNA (arrowheads). RNA-ISH. (g) Forestomach. In addition to numerous infiltrating leukocytes, a few squamous epithelial cells exhibit a nuclear signal for OvHV-2 RNA (arrowheads). RNA-ISH.

RNA-ISH in combination with IHC for leukocyte markers was used to characterize the target cell spectrum of OvHV-2 in the infected hamsters. This confirmed that circulating leukocytes carry viral RNA (Figs. 2d and 6) and hence spread the infection via viremia. Viral RNA was identified in both T-cells and monocytes in the blood, which was evidenced by hybridization in cells in hepatic sinusoids (Fig. 6a, c). Both T-cells and monocytes appeared to transport the virus into the extravascular tissue, as shown in the portal infiltrates (Fig. 6b, d). In addition, there was also strong evidence of viral RNA in occasional vascular endothelial cells (Fig. 6e). Closer investigation of the squamous epithelium of the tongues and forestomachs also revealed the presence of viral RNA in scattered squamous epithelial cells in inflamed areas (Figs. 3d, 4f, 6f, and g).

### Characterization of Hemolymphatic Tissues

**Figure 7.**
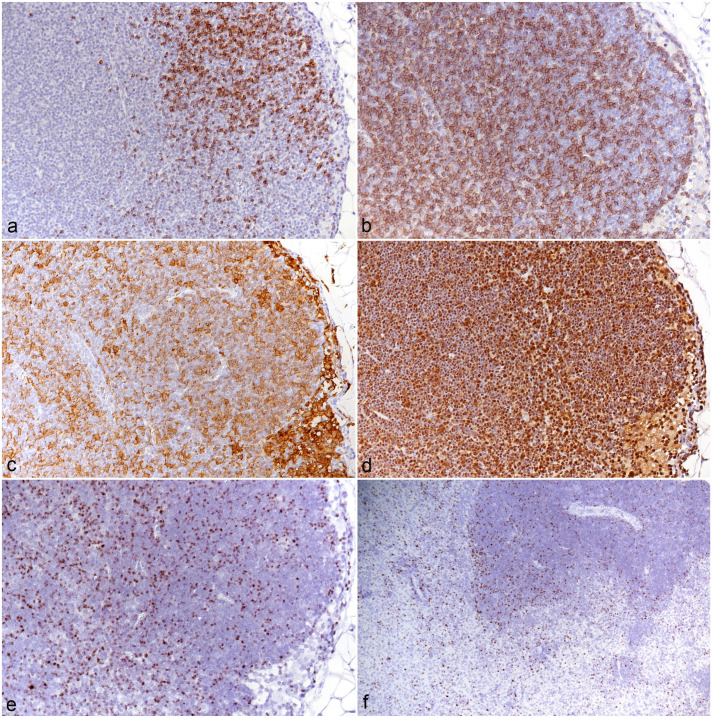
Mesenteric lymph node, case 10. (a) CD79a immunohistochemistry (IHC) for B-cells demonstrates a lack of distinct follicles. CD79a IHC. (b) CD3 IHC for T-cells highlights the expansion of the T-cell compartment. CD3 IHC. (c) IBA1+ macrophages expand the sinuses but are also abundant in areas populated by T-cells and B-cells. IBA1 IHC. (d) IHC for PCNA highlights abundant proliferating cells throughout the tissue. PCNA IHC. (e, f). Lymphatic tissue, case 10, RNA in situ hybridization for *Ov2.5*. (e) Numerous disseminated leukocytes harbor viral RNA. (f) Spleen. Numerous leukocytes in both red and white pulp harbor viral RNA.

In all animals in the main experiment, the cervical, mediastinal, and mesenterial lymph nodes were examined; from animals in the pilot experiment, individual lymph nodes were also included. All lymph nodes exhibited similar morphological features. The cortex and paracortex were generally poorly outlined due to a lack of distinct follicles and an expansion of the T-cell compartment (Fig. 7a, b; Supplemental Figures S5a, b). Macrophages (IBA1+) were abundant and were almost diffusely distributed (Fig. 7c; Supplemental Figure S5c). Similarly, cell proliferation which was most pronounced in follicle centers in the control animals (Supplemental Figure S5d) did not show any compartmental preference (Fig. 7d).

The spleens (n = 9) exhibited a variable composition. In some animals (cases 2-5 and 7), the white pulp was comprised of small primary follicles and small T-cell zones, and the red pulp was of low cellularity. In the remaining animals (cases 6 and 8-10), the follicles were of moderate size, and the T-cell zones were cell-rich and poorly delineated (Supplemental Figures S6a-d). The red pulp was of moderate to high cellularity, which appeared to be due to an increase in monocytes/macrophages (IBA1+) and T-cells (CD3+), among which were many proliferating (PCNA+) cells (Supplemental Figures S6c-h).

In both the lymph nodes and the spleen, numerous disseminated leukocytes harbored viral Ov2.5 RNA (Fig. 7e, f). Interestingly, whenever it was present in the section, the adjacent mesentery contained mild or moderate perivascular mononuclear (T-cells and macrophages) infiltrates that carried viral RNA.

The femoral and lumbar vertebral bone marrow (n = 6; main study) was cell-rich and appeared to contain more blastoid cells than in the age-matched control (Supplemental Figures S7a, b). It comprised a substantial number of individual or small groups of T-cells and numerous disseminated IBA1+ cells, whereas in the control animal, T-cells were absent and IBA1-positive cells were less abundant (Supplemental Figures S6c-f).

## Discussion

MCF is a gammaherpesvirus-induced sporadic disease of ruminants characterized by a variety of pathological changes affecting diverse organ systems but with a focus on blood vessels, skin, mucosa, and lymphatic tissues. The pathogenesis of these changes is not yet fully understood, indicating the need for suitable small animal models. So far, the rabbit has mainly been promoted as an appropriate model of MCF. However, a search of the literature indicates that it might not recapitulate all relevant processes of natural MCF in cattle and other ruminants.^4,10,13,16,34,42,65^

The present study reports a renewed attempt at the hamster model of SA-MCF, making use of in situ methodological approaches that were not available at the time when previous experimental infections were performed.^8^ It recapitulates and confirms the pathological effects of OvHV-2 in hamsters after intraperitoneal inoculation of lymphoid cells from infected animals and provides further insight into the viral target cell spectrum and potential pathomechanisms underlying disease,^8^ also highlighting relevant pathogenic aspects that have so far not been considered to a major extent.

Persistent elevated body temperature has been used as a parameter to monitor the clinical disease after experimental infection.^10,30,58,76^ In the present study, it also was a reliable clinical sign of the disease in the hamsters, similar to rabbits in previous studies,^34^ and was associated with viremia as determined by PCR on buffy coat cells from a first cohort of animals.

We inoculated hamsters intraperitoneally with lymphoid cells isolated from the spleen and lymph nodes of hamsters that had been experimentally infected with OvHV-2 in a prior experiment. Cells used in the initial study had been kept in liquid nitrogen for approximately 15 years. After thawing, live cells were selected and used for the inoculation at a dose of 2.2 × 10^5^ cells. Elevation of the body temperatures indicated the onset of clinical disease. However, the day of its onset post-inoculation varied between the 2 experiments and between individual animals, ranging from 29-41 dpi to 15-17 dpi in the first and second experiment, respectively. The reason might be that the cells for the second experiment were kept frozen for a much shorter time before use.

Using RNA-ISH, our in situ examinations revealed that both circulating T-cells and monocytes mediated the viremia. The first evidence that OvHV-2 is spread systemically not only by T-cells but also by monocytes was reported from experimental infections of sheep where some CD14+ monocytes were found to carry the virus.^45^ More recently, in our study on the arteritis in the rete mirabile of cattle with natural MCF, we found viral RNA in both circulating T-cells and monocytes.^63^ The results of the present study not only confirm this finding but also detected numerous infected T-cells and monocytes in lung capillaries and liver sinusoids, indicating their abundance in the circulating blood. Further studies would be required to determine whether these findings are associated with hematologic changes such as lymphocytosis or monocytosis.

Previous studies have suggested that in MCF the virus is carried by large granular lymphocytes or large lymphoblasts that are CD4+, CD8+, or CD4-/CD8- and grow in vitro without exogenous cytokines. These cells had an activated phenotype and consistent MHC-independent cytotoxicity,^7,10,64,71^ but it was not clear as to whether these were T-cells or NK cells.^71^ Similar to previous studies on naturally infected cattle with MCF,^54,63^ the present study cannot shed further light on the precise nature of the infected T-cells, as they likely all detected the fully assembled CD3 complex, which would not be expressed by NK cells.^48^ However, taken together, their results suggest that T-cells can be both lytically and latently infected.^54,63^

The literature frequently states that MCF is associated with hyperplasia of the lymphoid tissue, and some papers have even called it a “lymphoproliferative disease.”^71^ However, some publications took a more differentiated approach and reported the changes in the lymphatic tissues of cattle and bison with MCF to range from hyperplasia to depletion; the former appears to mainly affect the T-cell compartments.^21,51,76^ In both rabbits and hamsters, the most consistent reported morphological equivalent is an expansion of the T-cell compartments in lymph nodes and spleens.^4,13,16,34,65^ The present study confirmed this but also found evidence of expansion of the macrophage population and of increased proliferation of both (infected) T-cells and macrophages in spleen and lymph nodes of OvHV-2 infected hamsters. Whether the release of infected cells from these tissues then provides the cells for the observed inflammatory processes remains to be clarified. In a previous experimental study in rabbits, a proportion of cells in the hyperplastic lymphoid tissue could be identified as CD8+, followed by lesser numbers of CD4+ cells. Since cells positive for either marker together made up less than the cells expressing a pan T-cell marker, the authors concluded that, different from previous assumptions,^65^ the observed lymphoid hyperplasia was not due to expansion of the cytotoxic T-cell pool but to other, phenotypically altered lymphocytes.^4^ This represents another area of future research to clarify the pathogenesis of MCF.

The present study is the first to also examine the bone marrow of infected animals. It showed an increase in T-cells (CD3+) and IBA1+ cells (monocytes, possibly including some immature stages) among the hematopoietic cells. This would indicate the presence of infected cells and, potentially, their proliferation, which would suggest the bone marrow as a possible source of cell-associated viremia in animals with MCF.^2,5,6,73,75^ Unfortunately, we could not use our RNA-ISH approach on the bone marrow as the decalcification process destroys the RNA in the tissue (personal observation); however, this is an area that warrants further investigation and could clarify whether the virus can persist in the bone marrow.

The literature has provided variable information regarding the composition of the inflammatory infiltrates in both natural cases and animal models of MCF. An early light and electron microscopical study identified the vasculitis in MCF to be mediated by lymphocytes/lymphoblasts and fewer monocytes/macrophages.^40^ A subsequent study on 2 bovine MCF cases stated that macrophages made up more than half of the cells in the leukocyte infiltrates, accompanied by equal numbers or less T-cells (CD8+, CD4+).^49^ A few years later, the investigation of a calf and a bison with MCF found T-cells (CD3+; predominantly CD8+, no CD4+ cells) to dominate the infiltrates, accompanied by low numbers of monocytes/macrophages.^68^

A dominance of T-cells, with rare macrophages (CD14+) and B-cells, has also been reported in the infiltrates in infected rabbits,^4^ and in MCF-associated inflammatory processes in the brain of cattle.^24^ However, our recent studies on cattle and a goat with MCF confirmed that, indeed, macrophages make up a large proportion of the (vascular) infiltrates.^42,63^ This is well reflected by the results obtained from the cohort of experimentally OvHV-2 infected hamsters, suggesting this species as highly suitable to study the development of the inflammatory processes.

Our recent study into the inflammatory processes in and around the rete mirabile arteries in cattle with SA-MCF has not only shown that they are driven by circulating infected T-cells and monocytes, but also that OvHV-2 infects vascular endothelial cells and smooth muscle cells in the arterial media, suggesting that the virus plays a direct role in the recruitment of the inflammatory cells into the vascular wall and the perivascular compartment.^63^ A recent case report on SA-MCF in a goat supports this interpretation and indicated that endothelial cells become systemically infected, as it detected viral RNA (Ov2.5) and protein (OvHV-2 latency-associated nuclear antigen, oLANA) in lesional endothelial cells, and protein also in endothelial cells of unaffected vessels.^42^ In the present study, we detected virus by RNA-ISH for Ov2.5 in endothelial cells in portal veins.

Similar to natural SA-MCF in cattle and in the rabbit model,^13,16,34,65^ most inflammatory processes in infected hamsters were centered around blood vessels. They mainly represented perivascular infiltrates while vasculitis was observed but a less prominent feature. However, the hamsters consistently exhibited a valvular endocarditis affecting the left atrioventricular valves, which was mediated by (infected) T-cells and monocytes. Taken together, the findings suggest that the inflammatory processes develop due to circulating, activated, infected T-cells and monocytes that home to tissues and emigrate from vessels prone to leukocyte emigration. Distribution of the lesions might either depend on endothelial cell infection or the susceptibility of vessels to endothelial activation and interaction with activated leukocytes. In this respect, MCF might be similar to feline infectious peritonitis (FIP), a disease characterized by a phlebitis mediated and dominated by activated virus-infected monocytes. Interestingly, the phlebitis in FIP does not occur in all organs in affected cats but is rather stereotypically seen in leptomeninges, renal cortex (stellate veins), and eyes and less frequently in the lungs and liver.^31^ The fact that not only veins, where endothelial cells are known to better induce leukocyte adhesion than in arteries,^1,19^ are affected in MCF could indicate that MCF results in a more intense systemic endothelial cell activation than FIP.^1^ The consistent lesions in the atrioventricular valves in the hamsters in the present study is particularly interesting in this context. To our knowledge, virus-associated valvular endocarditis has so far only been reported with Coxsackie virus, an enterovirus, in individual patients, and in a mouse model of infection.^77^ Similarly, it is worth noting that the necrotic arteritis that is often seen in cattle^40,51,76^ was not observed in our hamster cohort. It is also less marked in bison with MCF,^51,66^ and might, again like in FIP,^32^ depend on the extent of viremia and systemic inflammatory response in individual animals.

The present study included a detailed histological investigation of a wide range of tissues, taking into account the distribution of lesions reported in both natural MCF and animal models. As shown in Supplemental Table S1, which provides an overview of the reported distribution of (inflammatory) lesions in cattle with MCF and in experimentally OvHV-2 infected rabbits and hamsters, a rather wide range of organs is affected by perivascular and/or interstitial infiltrates, including the liver (mainly portal areas), kidneys (interstitial infiltrates), lungs, and heart, with rather high consistency, whereas involvement of other organs is less frequent. In cattle, the brain is often involved, which is characterized by nonsuppurative meningoencephalitis with or without lymphohistiocytic and, less often, necrotic vasculitis.^21,24^ We made similar observations in the hamster cohort, whereas in rabbits, the brain and/or meninges seem not to be affected.^13^ In the present study, the histological examination of the spinal cord revealed a mild leukomyelitis and leptomeningitis, with periganglioneuritis of the spinal ganglia in all examined animals. The infiltrate had the same composition as elsewhere and included virus-infected cells; it was associated with evidence of myelin degradation (presence of myelinophages), microgliosis and satellitosis. In most examined cases, the infiltrate also affected the sciatic nerve, and the biceps femoris muscle in some cases. Whether such inflammatory processes also occur with natural infections and in the rabbit is not known as these tissues have not been reported to be examined histologically. Taken together, the findings indicate a more widespread distribution of the inflammatory processes than generally reported. However, no evidence was found that OvHV-2 infects parenchymal cells in the brain or muscle cells.^24^

The present study laid a focus on the presence and type of changes in the skin and the mucosa of the alimentary tract, taking into account that MCF is associated with rather consistent lesions at these sites in cattle and bison,^66,76^ and that we recently found erythema multiforme-like lesions in a goat with MCF.^42^ In cattle with MCF, the most detailed information on such lesions is available from an older study that investigated oral mucosa and esophagus in detail by light and electron microscopy.^40^ This characterized the (sub)epithelial infiltrates as mononuclear, dominated by lymphocytes/lymphoblasts, with fewer macrophages that increased in number with the severity of the lesions. Lymphocytes were found adjacent to epithelial cells and there was evidence of phagocytosis of degenerate epithelial cells. The authors also described acantholysis and necrosis of epithelial cells.^8,40,50^ The recently reported case of erythema multiforme-like skin lesions in a goat with SA-MCF described ulceration, transepidermal apoptosis with satellitosis, interface dermatitis, folliculitis, and dermal arteritis and found viral RNA (Ov2.5) not only in the nucleus of infiltrating leukocytes and vascular endothelial cells but also in keratinocytes, suggesting that a cell-mediated immune response against the virus might at least contribute to the development of the lesions.^42^ The present study consistently observed similar, partly ulcerative lesions on tongue and forestomachs of the infected hamsters, comparable to an older study.^8^ Like the skin of the goat with natural SA-MCF, the affected squamous epithelium contained epithelial cells dying by apoptosis.^42^ It is not unlikely that the cell death described in the earlier paper also represents apoptosis.^40^ Indeed, a review paper on the pathology of SA-MCF has shown images from the urinary bladder and oral mucosa of an affected bison with apoptosis of epithelial cells.^51^ The older detailed study also showed that epithelial lesions are widespread in cattle and affect the entire gastrointestinal tract, the conjunctiva, the respiratory tract, various glandular ducts, the urinary bladder, and the choroid plexus, with degenerative epithelial changes exclusively at sites of inflammatory infiltration.^40,41^ Despite the presence of vasculitis and/or perivascular infiltrates in the deeper lamina propria, and the earlier assumption that the lesions could result from vascular disruption,^62^ the authors concluded that due to the lack of substantial infarcts and vascular thrombi, ischemia does likely not play a major role in the pathogenesis of the epithelial lesions. Instead, they saw substantial similarity to lesions seen in graft versus host disease.^40^ The results of the present study support this interpretation, considering also that a previous report mentioned a dominance of macrophages in vascular and epithelial lesions in 2 natural cases of MCF in cattle.^49^ However, while we detected apoptotic cells in lesional epidermis/squamous epithelium in both the goat with MCF and our hamsters, we did not find morphological evidence that latently infected epithelial cells underwent apoptosis.^42^ This is in line with our recent findings in MCF vasculitis in cattle,^63^ and another study that did not even report damage in lytically infected cells.^54^ Hence, any virus-induced changes, whether functional or phenotypic, appear not to present as direct cytopathic effects. Whether the epithelial cells were latently infected or even promoted lytic infection could not be determined, as Ov2.5, the target of our RNA-ISH that encodes a protein similar to ovine interleukin-10, is expressed during both viral phases.^3,29^ It is also not clear in which way infection of the infiltrating leukocytes contributes to this scenario. Does it require infected, activated T-cells and monocytes/macrophages to home in??? the tissue (i.e., squamous epithelium) and carry infection to epithelial cells, or is it the infection of the epithelium that attracts the leukocytes? Does the (accidental) recruitment of virus-infected T-cells and monocytes through the vasculitis foster or potentiate the mucosal/epidermal graft versus host disease-like processes?

In the intestine, we observed diffuse mucosal infiltration by virus-infected T-cells and macrophages, with villus blunting and fusion, but there was no evidence of viral infection of enterocytes. Similarly, we did not detect viral RNA in respiratory or alveolar epithelial cells. This suggests a selective epithelial tropism of the virus, at least in hamsters. It remains open whether the strong infiltration of the intestinal mucosa occurs because this compartment contains abundant leukocytes and in particular residential T-cells, rendering it prone to more intense leukocyte recruitment. In experimentally (BJ 1035 cells) infected rabbits, RNA-ISH provided evidence of viral replication in epithelial cells in the appendix.^46^ In this study, OvHV-2 *ORF63* mRNA was detected, and *ORF63* is predicted to code for a viral tegument protein.^46^ However, since the study did not detect any other cells with an *ORF63* mRNA signal in any other examined tissues (mesenteric lymph node, lung, spleen, liver, and kidney), it is difficult to compare it with other studies, including ours.^54^

A previous study used a mouse monoclonal antibody (15-A) against a conserved epitope of MCF viruses, binding a 45 kDa protein of a larger glycoprotein complex,^38^ on cattle with SA-MCF and detected OvHV-2 antigen in the cytoplasm of a wide range of epithelial cells (renal tubules, degenerating bile ducts, and small intestinal degenerating crypts), in cells with macrophage morphology in the mucosa of the small intestine with inflammatory infiltration and in the mesenteric lymph node, and in endothelial cells in renal medullary capillaries, but not in the brains despite the presence of meningoencephalitis and vasculitis.^22^ The epithelium of the respiratory tract was not found to be positive, although it is consistently infected in the acute phase of most gammaherpesvirus infections in their natural hosts.^25,74^

The present study cannot yet address the key question regarding the pathogenesis of MCF, as it did not investigate the trigger for the proliferation and activation of infected leukocytes and hence the inflammatory processes. It can also not provide answers to which extent the virus targets vascular endothelial cells and (squamous) epithelia and whether the recently suggested even broader target cell spectrum is of major relevance for the development of the disease.^22^ However, it offers the hamster as a suitable experimental host for studies on the pathogenesis of SA-MCF; with its help, it should be possible to further investigate the interplay between virus and host cells and between infected cells, with particular emphasis on a potential graft versus host reaction.

## Supplemental Material

sj-pdf-1-vet-10.1177_03009858251315115 – Supplemental material for The golden Syrian hamster (Mesocricetus auratus) as a model to decipher relevant pathogenic aspects of sheep-associated malignant catarrhal fever Supplemental material, sj-pdf-1-vet-10.1177_03009858251315115 for The golden Syrian hamster (Mesocricetus auratus) as a model to decipher relevant pathogenic aspects of sheep-associated malignant catarrhal fever by Rosalie Fabian, Eleanor G. Bentley, Adam Kirby, Parul Sharma, James P. Stewart and Anja Kipar in Veterinary Pathology
