# Supplementary material for: The golden Syrian hamster (Mesocricetus auratus) as a model to decipher relevant pathogenic aspects of sheep-associated malignant catarrhal fever
Source: Vet Pathol. 2025 Feb 11;62(5):697–713. doi: 10.1177/03009858251315115 (PMC12314217; doi:10.1177/03009858251315115)
Supplement: sj-pdf-1-vet-10.1177_03009858251315115 – Supplemental material for The golden Syrian hamster (Mesocricetus auratus) as a model to decipher relevant pathogenic aspects of sheep-associated malignant catarrhal fever [file sj-pdf-1-vet-10.1177_03009858251315115.pdf]

Supplemental Materials

The golden Syrian hamster (*Mesocricetus auratus*) as a model to decipher relevant pathogenic aspects of sheep-associated malignant catarrhal fever

Rosalie Fabian, Eleanor G Bentley, Adam Kirby, Parul Sharma, James P. Stewart, Anja Kipar

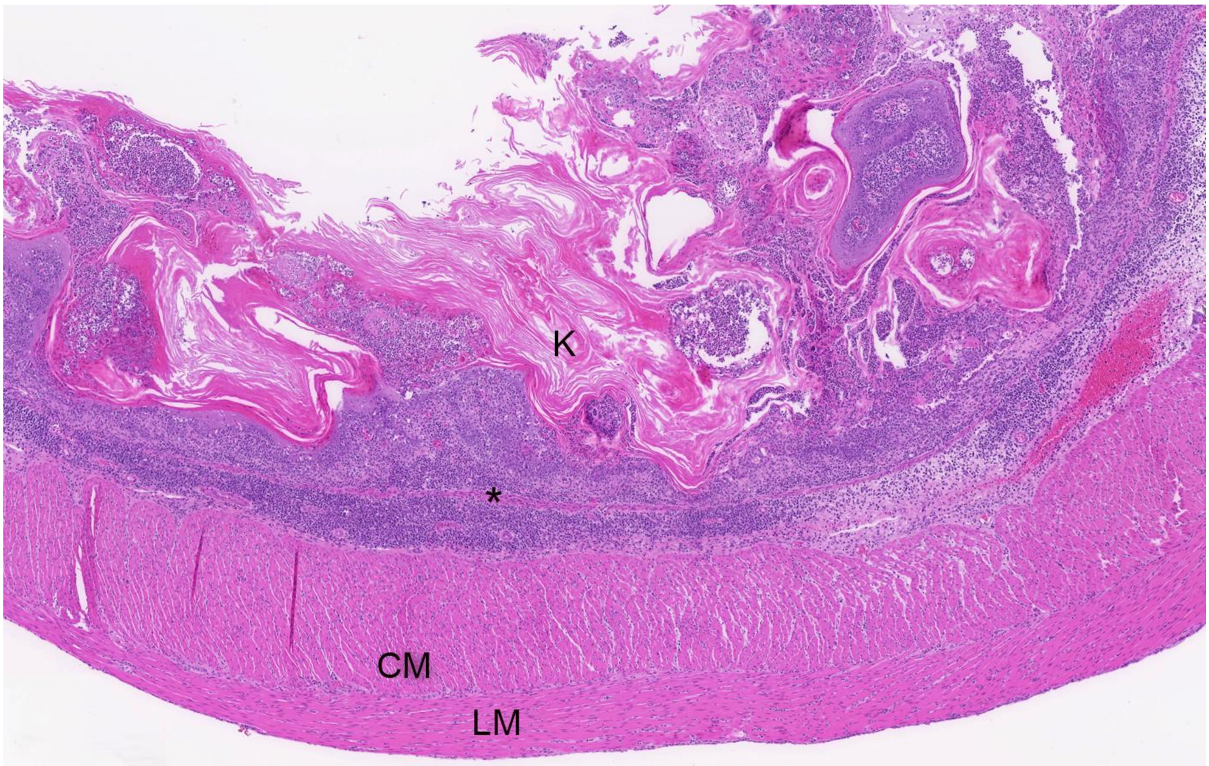

Figure S1. Forestomach, case 3. Overview of an area with extensive serocellular crust formation and marked mucosal and submucosal mononuclear cell infiltration. K, keratin layer; CM, circular muscle layer; LM, longitudinal muscle layer; asterisk, muscularis mucosae. Hematoxylin and eosin.

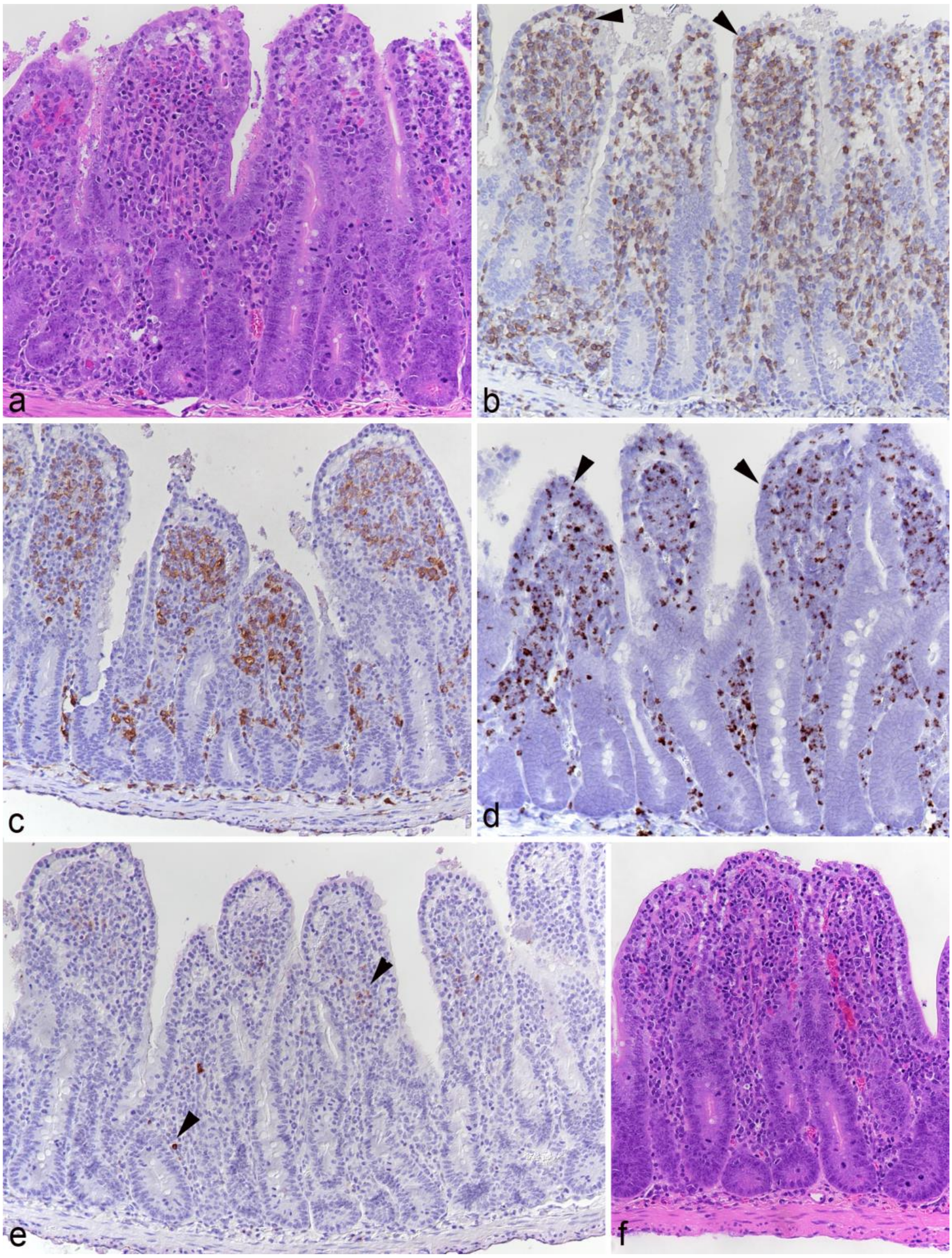

Figure S2. Small intestine, case 3. **a.** Diffuse thickening of villi due to marked diffuse leukocyte infiltration of the mucosa. Hematoxylin and eosin (HE). **b.** The leukocyte infiltrate is dominated by CD3+ T-cells that also infiltrate the epithelial layer (arrowheads). CD3 Immunohistochemistry (IHC). **c.** IBA1+ macrophages are the second most abundant leukocyte population in the mucosal infiltrate. IBA1 IHC. **d.** Abundant infiltrating leukocytes, including some of those infiltrating the epithelial layer (arrowheads) are positive for viral RNA (*Ov2.5*). RNA-in situ hybridization. **e.** Among the infiltrating leukocytes in the mucosa are scattered CD79a+ B-cells and plasma cells (arrowheads). CD79a IHC. **f.** The intense mucosal infiltrate is associated with blunting and fusion of the thickened villi. HE.

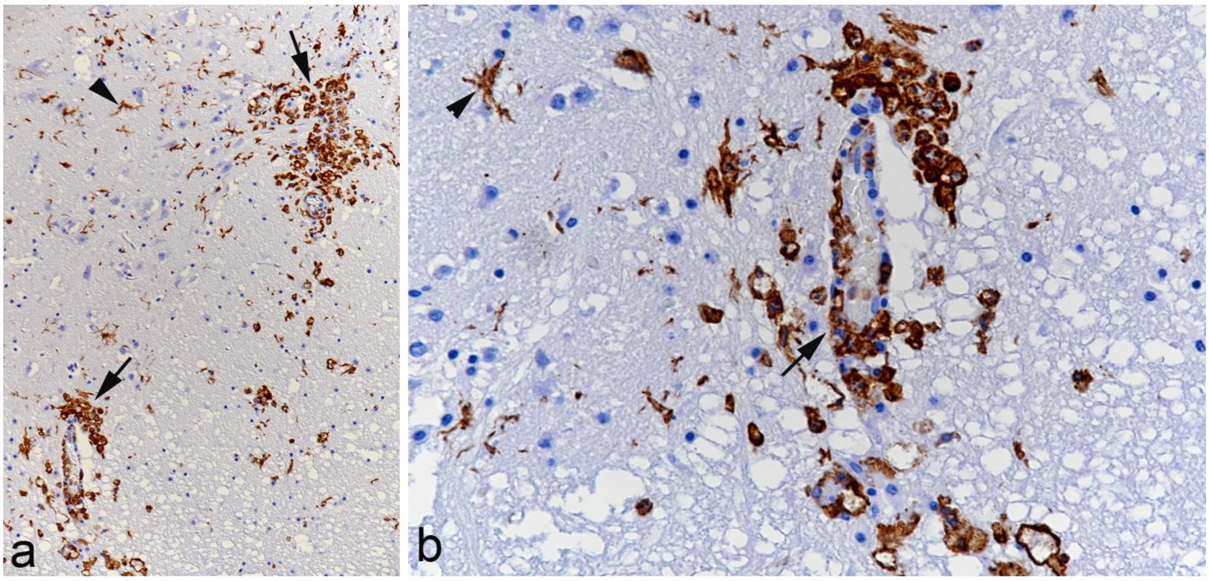

Figure S3. Lumbar spinal cord, case 7. Immunohistochemistry for IBA1. **a.** There are focal areas of microgliosis (arrows) and activated microglial cells (arrowhead) in the parenchyma. **b.** Higher magnification of a vessel with surrounding parenchyma confirms monocyte emigration, perivascular macrophage infiltration (arrow), and the presence of activated microglial cells (arrowhead).

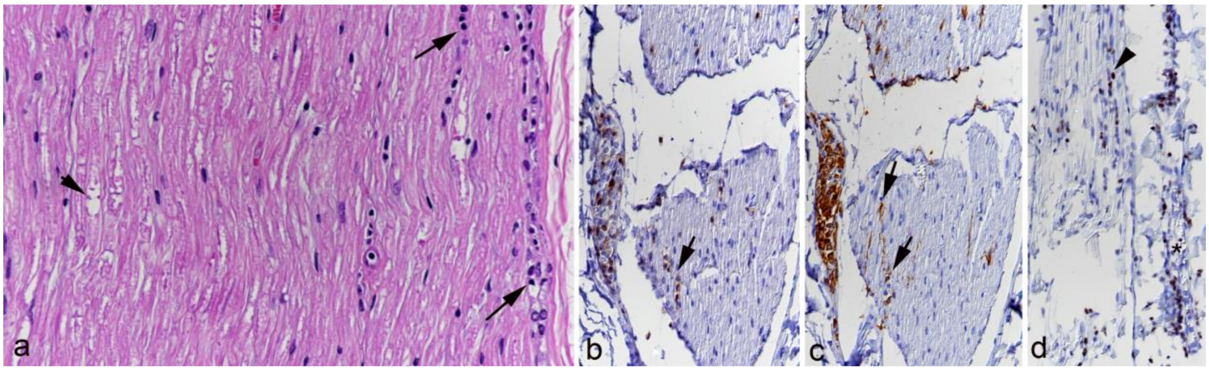

Figure S4. Sciatic nerve, case 10. **a.** There are individual degenerate nerve fibers (arrowhead) and a few infiltrating leukocytes (arrows). Hematoxylin and eosin. **b.** CD3+T-cells infiltrating between nerve fibers (arrow). CD3 immunohistochemistry (IHC). **c.** IBA1+ macrophages infiltrating between nerve fibers (arrows). IBA1 IHC. **d.** Infiltrating leukocytes harbor OvHV-2 RNA (Ov2.5) (arrowhead). RNA-in situ hybridization.

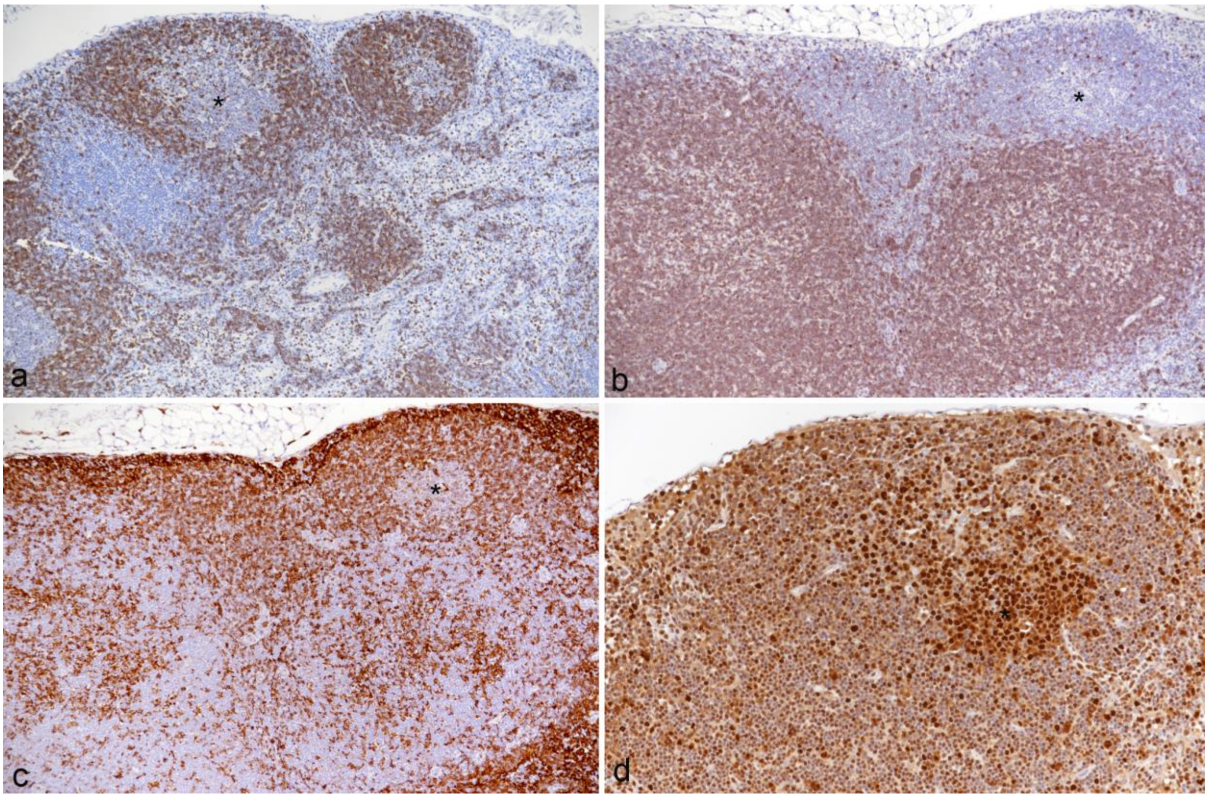

Figure S5. Mesenteric lymph node, control animal. The asterisk highlights a follicular germinal center. **a.** CD79a immunohistochemistry (IHC) for B-cells highlights several distinct follicles comprising the cortex. CD79a IHC. **b.** CD3 IHC for T-cells highlights a well-defined paracortex (T-cell compartment). CD3 IHC. **c.** IBA1 + macrophages are mainly seen in sinuses and in the cortex, they are less numerous in the paracortex. IBA1 IHC. **d.** IHC for PCNA highlights pronounced proliferation in the germinal center of a follicle. PCNA IHC.

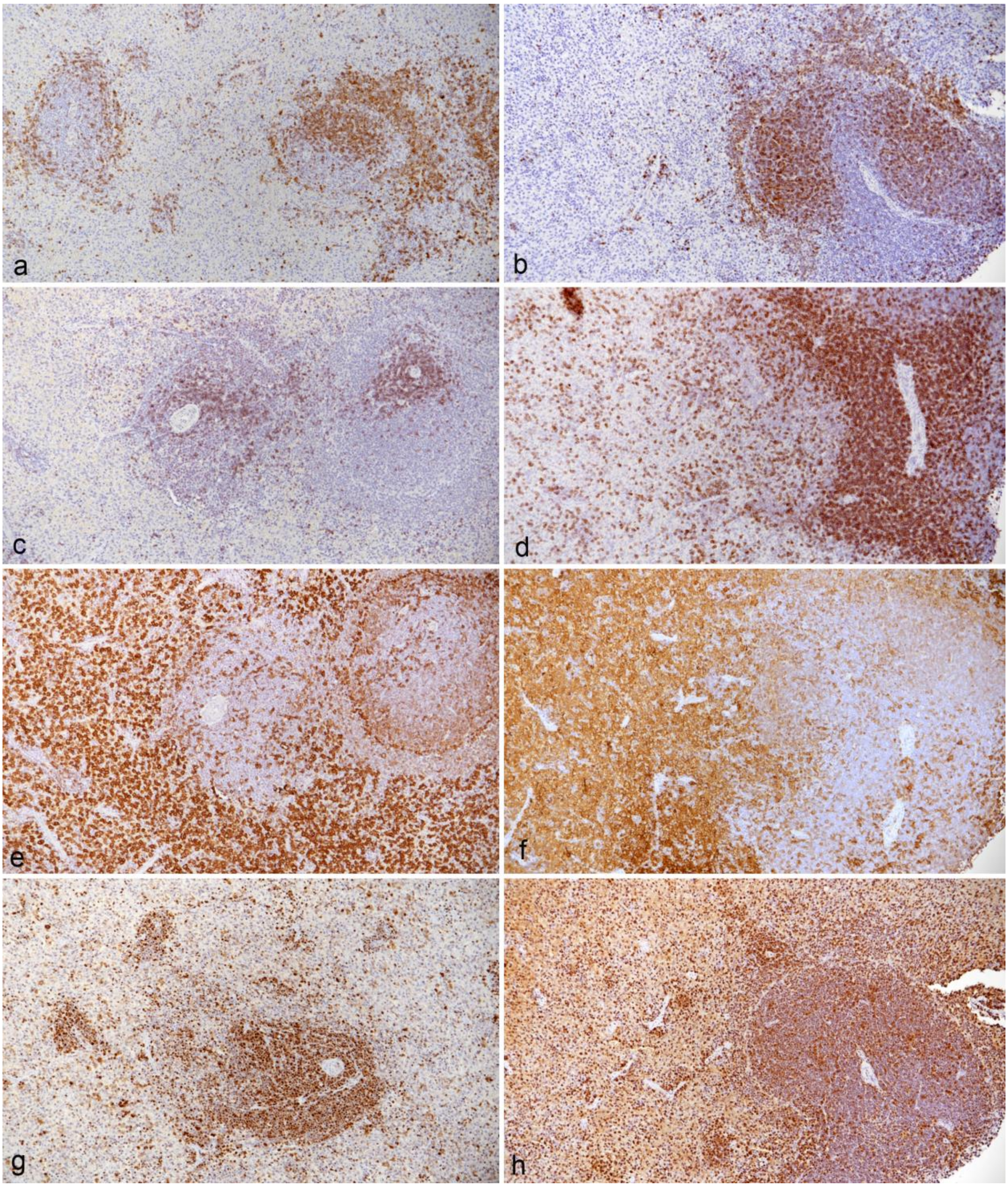

Figure S6. Spleen, control animal and infected case 10. **a, b.** CD79a immunohistochemistry (IHC) for B-cells identifies small follicles in both the control (**a**) and the infected (**b**) animal. **c, d.** CD3 IHC for T-cells. **c.** In the control animal, T-cell zones are small. T-cells are present in low numbers in the red pulp. **d.** In the infected hamster, T-cell zones are of moderate size, and there are numerous T-cells in the red pulp. **e, f.** IBA1+ monocytes/macrophages are abundant in the red pulp in the control animal (**e**) and in the infected animal (**f**). **g, h.** IHC for PCNA. **g.** The control hamster exhibits abundant proliferating cells in the white pulp; in the red pulp they are present in low numbers. **h**. In the infected animal, proliferating cells are abundant in both red and white pulp.

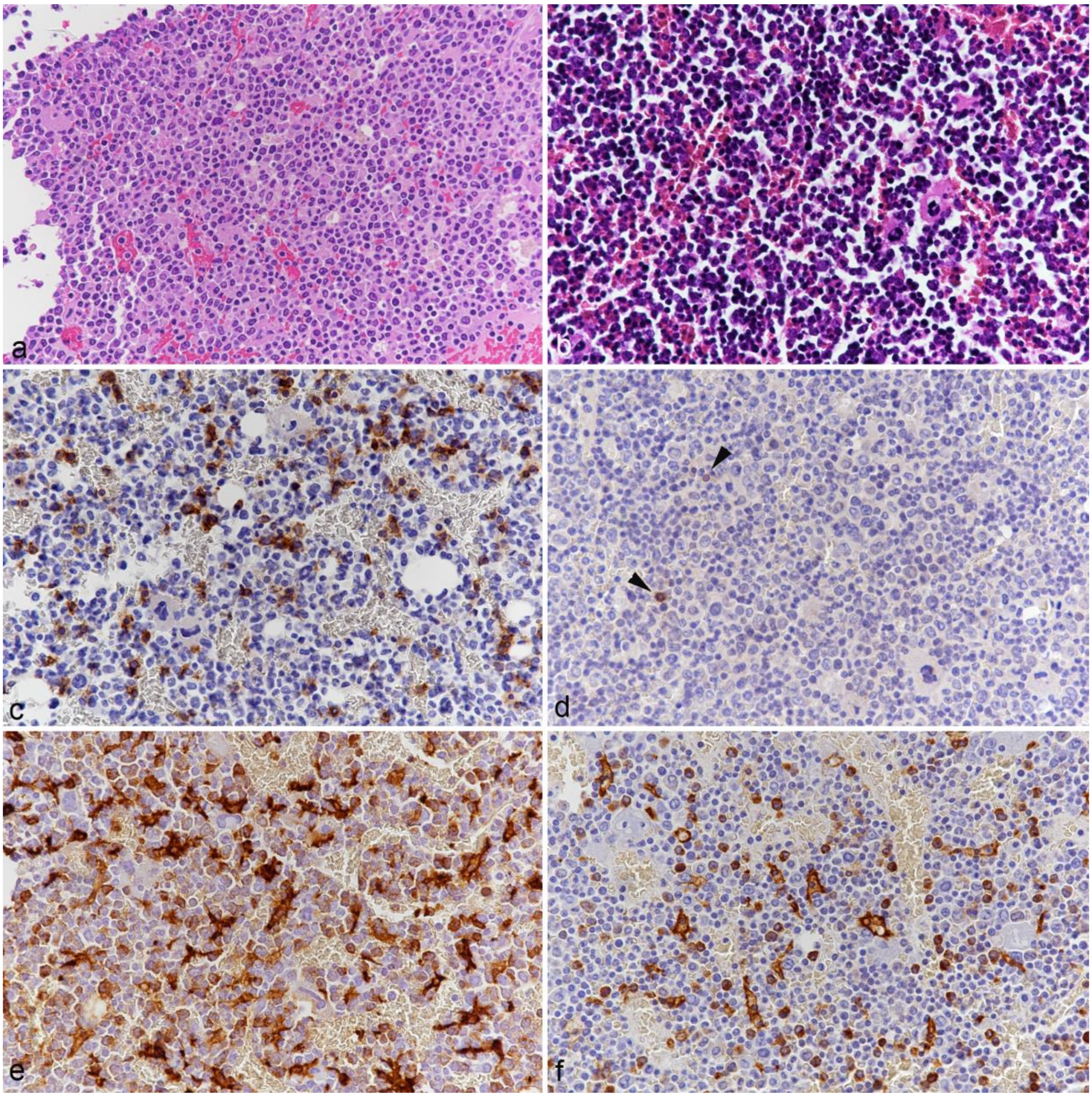

Figure S7. Femoral bone marrow. Left column (a, c, e): case 8; right column (b, d, f): control animal. **a, b.** Hematoxylin and eosin. The bone marrow is cell rich. Overall, there appear to be more blastoid cells in the infected animals (a) than in the control animal (b). **c, d.** CD3 immunohistochemistry (IHC) for T-cells. (c) In the infected animal, there are numerous individual or small groups of T-cells. (d) In the control animal, T-cells (arrowheads) are rare. **e, f.** IHC for IBA1. (e) Numerous positive cells are seen disseminated in the infected animal. (f) In the control animal, the number of positive cells is far lower.

Supplemental Table S1. Pathological changes reported in systematic studies in natural sheep-associated malignant catarrhal fever (SA-MCF) in cattle or summarized in a recent textbook,[16] in the rabbit model of SA-MCF, and in the hamster model as observed in the present study, complemented by findings reported in a previous publication.[2]

Organ/tissue	Natural MCF (cattle)	Rabbit model	Hamster model
Vessels and heart	Vessels: disseminated mononuclear vasculitis and pv accumulations[8,12,15,16]<br>Heart: lymphoplasmacytic myocarditis[8]	Vessels: general arteriolitis-phlebitis in bronchial circulation[4,10]<br>Heart: occasional interstitial LC accumulations[6]	Vessels: vasculitis in occasional portal veins (liver) and of vessels in brain parenchyma with encephalitis, widespread pv infiltrations (TM[b])[c]<br>Heart: variable degree of endocarditis and myocarditis;[2] valvular endocarditis (AV valves), occasional parietal endocarditis and myocardial interstitial infiltrates (TM[b])[c]
Alimentary tract mucosa	Erosive/ulcerative lesions and/or lymphoplasmacytic inflammatory infiltrates: tongue, gingiva, oral mucosa, esophagus, forestomachs, abomasum, intestines[8,11,13,15]	Esophagus: focal subepithelial mononuclear lymphoid cell accumulations[a] associated with ballooning degeneration of the epithelium[14]<br>Intestines: information limited to appendix in areas of lymphoid structures (see below)	Oral mucosa: inflammatory processes tongue, cheek pouches, hard palate[2]; subepithelial infiltrates in tongue[d] (TM[b])[c]<br>Esophagus: subepithelial infiltrates (TM[b])[c]<br>Non-glandular forestomach: acantholysis and hyperkeratosis[2]; erosive/ulcerative proventriculitis[d], marked submucosal infiltration (TM[b])[c]<br>Stomach: mild/moderate infiltration of the mucosa (TM[b])[c;2]<br>Small intestine: marked diffuse mucosal infiltration (TM [b])[c;2]<br>Caecum: ulcerative lesions[2]
Liver	Pv mononuclear infiltration in portal areas[8,16]	Peribiliary and perivascular lymphoid cell accumulations[a14]; periportal lymphoid cell accumulations (mainly T cells)[1,3]; portal hepatitis (large LC, fewer macrophages, plasma cells and heterophils), arteritis and phlebitis, biliary hyperplasia, focal areas of hepatocellular necrosis[6]; (peri)cholangitis, portal phlebitis-arteritis, hepatic necrosis, granulomatous lesions[10]	Moderate portal infiltrates (TM[c])[2]
Lymphatic tissue, bone marrow	Lymph nodes: variable; hyperplasia in T cell dependent areas in interfollicular	Lymph nodes (MLN): expansion of the paracortex[14]/interfollicular zones (T cells)[3]; marked hyperplasia of cortex, paracortex and lymphoid	Lymph nodes: lymphadenitis and hyperplasia[2]; expansion of T cell compartment and macrophage population, with proliferation of cells[c]

	cortical and paracortical zones, depletion[8,16]<br>Spleen: variable, ranging from lymphoid cell hyperplasia in PALS to depletion[8,16]	follicles, focal necrosis (mainly T cells);[1] mild expansion of paracortex (and cortex) by large LC;[6] diffuse lymphadenitis with progression to granulomatous lymphadenitis[10]<br>Lymphoid tissue (appendix): hyperplasia of T cell areas, necrosis;[14] marked hyperplasia of lymphoid follicles and interfollicular areas (mainly T cells), necrosis of lymphoid follicles[1]<br>Spleen: hyperplasia of PALS and red pulp (mainly T cells);[1,3] mild increase in large LC in PALS[6]	Spleen: prominent PALS;[2] variable expansion of T cell zones, increased cellularity of red pulp (TM[b])[c]<br>Bone marrow: cell rich; with substantial numbers of T cells and numerous monocytes (Iba1 positive cells)[c]<br>Thymus: premature involution[2]
Central and peripheral nervous system, eyes	Brain: nonsuppurative meningoence-phalitis with(out) lymphohistiocytic vasculitis and/or necrotic vasculitis[8,9]<br>Spinal cord, peripheral nerves: no lesions described/not examined<br>Eyes: lympho(plasma)cytic conjunctivitis, keratitis, iridocyclitis, uveitis[8,12]	Brain: no evidence of meningitis and/or encephalitis[4]<br>Spinal cord, peripheral nerves: not examined<br>Eyes: lymphoid hyperplasia in palpebral conjunctiva;[3] gross evidence of keratoconjunctivitis;[10] no evidence of keratoconjunctivitis[4]	Brain: mononuclear leptomeningitis and encephalitis (TM[c]); mild lymphoid meningitis[2]<br>Spinal cord: lymphohistiocytic leukomyelitis, leptomeningitis and periganglionitis (TM[b])[c]<br>Sciatic nerve: mild focal neuritis and perineuritis (TM[b])[c]<br>Eyes: mononuclear infiltration of conjunctival lamina propria with acanthosis and hyperkeratosis at conjunctival-epidermal junction[2]
Respiratory tract	Nose: (ulcerative) lymphoplasmacytic rhinitis[7,8]<br>Airways: erosive tracheobronchitis[16]<br>Lungs: interstitial pneumonia, bronchopneumonia[8]	Nose: no lesions described (not examined)<br>Trachea: lymphoid cell accumulations[a14]<br>Lungs: perivascular and peribronchial/-bronchiolar lymphoid cell accumulations (mainly T cells);[1] interstitial pneumonia with intralesional phlebitis-arteritis[10]	Nose: not examined<br>Trachea: focal mononuclear infiltrates[2]<br>Lungs: arteritis and focal interstitial lymphoid infiltrates;[2] increase in TM[b] in capillaries, some leukocyte emigration and perivascular accumulation[c]
Urinary tract	Kidneys: pv mononuclear infiltrations in cortex, lymphocytic interstitial nephritis;[8,16]<br>Urinary bladder: (ulcerative) lympho-plasmacytic or hemorrhagic cystitis[8,11]	Kidneys: lymphoid cell accumulations[a];[14] frequent perivascular LC accumulations in cortex (mainly T cells);[1] occasional interstitial LC accumulations and occasional vasculitis.[6]<br>Urinary bladder: pv LC accumulations[6]	Kidney: focal interstitial infiltrations and/or focal pyelitis (TM[b]) [c]<br>Urinary bladder: ulcerative cystitis;[2] mild to moderate heterophilic cystitis[c]
Skin	Ulcerative/necrotic dermatitis in several locations; lichenoid infiltration in upper dermis, stretching into epidermis[5,16]	Not examined	Occasional mononuclear infiltrates in dermis, degeneration of basal cells and hyperkeratosis of overlying epidermis;[2] no changes observed[c]
Other locations	Variable degree of mononuclear infiltration beneath and between epithelial cells in respiratory tract, entire gastrointestinal tract, biliary epithelium,	Occasional interstitial LC accumulations in adrenal glands, thyroid glands, pancreas, tongue, trachea, sclera[6]	Skeletal muscles: occasional necrosis and interstitial mononuclear infiltration (diaphragm and cremaster);[2] very mild interstitial, pv leukocyte infiltrates[c]

	glandular ducts, choroid plexus;[11] lymphoplasmacytic sinusitis, thyroiditis[8]

Abbreviations: AV, atrioventricular; LC, lymphocyte(s); MLN, mesenteric lymph nodes; PALS, periarteriolar lymphoid sheaths; pv, perivascular

[a]The lymphoid cells in non-lymphoid tissues were CD43+ T cells, as determined by immunohistochemistry.
[b]TM: The infiltrate is comprised of T cells (CD3+) and macrophages (IBA1+) of which a proportion is infected, as shown by RNA-ISH for Ov2.5.
[c]Results obtained in the present study
[d]The infiltrates are accompanied by apoptosis of epithelial cells.

References:

1. Anderson IE, Buxton D, Campbell I, et al. Immunohistochemical study of experimental malignant catarrhal fever in rabbits. *J Comp Pathol*. 2007;136(2–3):156–166.
2. Buxton D, Jacoby RO, Reid HW, Goodall PA. The pathology of "sheep-associated" malignant catarrhal fever in the hamster. *J Comp Pathol*. 1988;98(2):155–166.
3. Buxton D, Reid HW, Finlayson J, Pow I. Pathogenesis of "sheep-associated" malignant catarrhal fever in rabbits. *Res Vet Sci*. 1984;36(2):205–211.
4. Cunha CW, O'Toole D, Taus NS, Shringi S, Knowles DP, Li H. A rabbit model for sheep-associated malignant catarrhal fever research: from virus infection to pathogenesis studies and vaccine development. *Curr Clin Microbiol Rep*. 2019;6(3):148–155.
5. David D, Dagoni I, Garazi S, Perl S, Brenner J. Two cases of the cutaneous form of sheep-associated malignant catarrhal fever in cattle. *Vet Rec*. 2005;156(4):118–120.
6. Gailbreath KL, Taus NS, Cunha CW, Knowles DP, Li H. Experimental infection of rabbits with ovine herpesvirus 2 from sheep nasal secretions. *Vet Microbiol*. 2008;132(1–2):65–73.
7. Headley SA, Oliveira TES, Li H, et al. Immunohistochemical detection of intralesional antigens of Ovine Gammaherpesvirus-2 in cattle with sheep-associated malignant catarrhal fever. *J Comp Pathol*. 2020;174:86–98.
8. Headley SA, de Oliveira TES, Cunha CW. A review of the epidemiological, clinical, and pathological aspects of malignant catarrhal fever in Brazil. *Braz J Microbiol*. 2020;51(3):1405.

9. Hierweger MM, Boujon CL, Kauer R V., Meylan M, Seuberlich T, Oevermann A. Cerebral Ovine Herpesvirus 2 infection of cattle is associated with a variable neuropathological phenotype. *Vet Pathol*. 2021;58(2):384–395.

10. Li H, Cunha CW, Gailbreath KL, et al. Characterization of ovine herpesvirus 2-induced malignant catarrhal fever in rabbits. *Vet Microbiol*. 2011;150(3–4):270–277.

11. Liggitt HD, Demartini JC. The pathomorphology of malignant catarrhal fever. II. Multisystemic epithelial lesions. *Vet Pathol*. 1980;17(1):73–83.

12. Liggitt HD, DeMartini JC, McChesney AE, Pierson RE, Storz J. Experimental transmission of malignant catarrhal fever in cattle: gross and histopathologic changes. *Am J Vet Res*. 1978;39(8):1249–1257.

13. O'Toole D, Taus NS, Montgomery DL, Oaks JL, Crawford TB, Li H. Intra-nasal inoculation of American bison (Bison bison) with ovine herpesvirus-2 (OvHV-2) reliably reproduces malignant catarrhal fever. *Vet Pathol*. 2007;44(5):655–662.

14. Schock A, Reid HW. Characterisation of the lymphoproliferation in rabbits experimentally affected with malignant catarrhal fever. *Vet Microbiol*. 1996;53(1–2):111–119.

15. Schultheiss PC, Collins JK, Spraker TR, DeMartini JC. Epizootic malignant catarrhal fever in three bison herds: differences from cattle and association with ovine herpesvirus-2. *J Vet Diagn Invest*. 2000;12(6):497–502.

16. Uzal FA, Plattner BL, Hostetter JM. Alimentary System. In: Grant Maxie M, ed. Jubb, Kennedy & Palmer's Pathology of Domestic Animals: Volume 2. 6th ed. Elsevier; 2015.

Supplemental Table S2. Antibodies, antigen retrieval, and detection methods used in immunohistology

Antigen	Antibody (clone)	Dilution (incubation)	Pretreatment	Detection method
CD3	Rabbit mAb (SP7)[a]	1:400 (1h, 37 °C)	EDTA[b]	Discovery Chromo Map (OmniMAP anti-Rabbit)[b]
CD79a	Mouse mAb (MH57)[c]	1:1,000 (1h, RT)	EDTA	EnVision Mouse[d]
IBA1	Rabbit pAb[e]	1:1,000 (1h, RT)	Citrate	EnVision Rabbit[d]
PCNA	Mouse mAb (PC10)[d]	1:800 (ON, 4 °C)	Citrate	MACH-4[f]
Cleaved caspase 3	Rabbit mAb[g]	1:200 (ON, 4 °C)	Citrate	EnVision Rabbit[d]

Abbreviations: IBA1, ionized calcium binding adaptor molecule 1; pAb, polyclonal antibody; mAb, monoclonal antibody; ON, overnight; PCNA, proliferating cell nuclear antigen; RT, room temperature

EDTA: 20 min incubation in EDTA buffer (pH9; Dako/Agilent) in a pressure cooker at 98 °C.

Citrate: 20 min incubation in citrate buffer (pH 6; Dako/Agilent) in a pressure cooker at 98 °C.

[a] Bioscience
[b] Ventana/Roche
[c] Bio-Rad
[d] Dako/Agilent
[e] Wako
[f] Biocare Medical
[g] Cell Signaling Technology

Supplemental Table S3. Pilot study, information on the day of euthanasia post-challenge, body temperature at euthanasia, and organs affected by the histologically observed leukocyte infiltrates. The histological changes are described in the main manuscript; here only those organs are listed that showed leukocyte infiltrates (i.e. T-cell and macrophage infiltrates with viral mRNA signal).

Animal	Euthanasia (dpi)	Body temperature	Affected organs
1	29	37.4 °C	Lungs
2	33	37.4 °C	Tongue, stomach, SI, liver, lungs, kidneys, brain
3	41	37.8 °C	Tongue, stomach, SI, liver, lungs, kidneys, brain
4	41	36.2 °C	Tongue, stomach, SI, liver, lungs, brain
5	17	31.7 °C	Tongue, stomach, SI, liver, heart, lungs, kidneys, brain, SC
6	17	38.0 °C	Tongue, stomach, SI, liver, heart, kidneys, brain, SC, SN
7	17	38.0 °C	Tongue, stomach, SI, liver, heart, kidneys, brain, SC, SN
8	15	37.5 °C	Tongue, stomach, SI, liver, heart, kidneys, brain, SC, SN
9	17	37.7 °C	Tongue, stomach, SI, liver, heart, kidneys, brain, SC, SN
10	17	37.8 °C	Tongue, stomach, SI, liver, heart, kidneys, brain, SC, SN

dpi, days post-inoculation
Initial study (cases 1-4): From animal 1, only the lungs were examined histologically; from cases 2-4, skin (dorsum), tongue, stomach, small intestine (SI), liver, spleen, lungs, kidneys, and the brain were examined.
Second study (cases 5-10): From all animals, tongue, esophagus, stomach, small intestine (SI), liver, heart, trachea, lungs, kidneys, brain, lumbar spinal cord (SC), right sciatic nerve (SN) and *Musculus biceps brachii* as well as the spleen and the cervical, mediastinal and mesenteric lymph nodes were examined.
